# Fluorinated poly(aryl ether)s containing difluoromethylene and tetrafluoroethylene moieties

**DOI:** 10.1039/d5ra08064a

**Published:** 2025-12-03

**Authors:** Ying Chang, Adi Avi-Izak, Timothy M. Krentz, Matthew Ravalli, Chang Y. Ryu, Chulsung Bae

**Affiliations:** a Department of Chemistry and Chemical Biology, Rensselaer Polytechnic Institute Troy New York 12180 USA baec@rpi.edu; b Department of Chemistry, University of Nevada Las Vegas 4505 Maryland Parkway, Box 454003 Las Vegas Nevada 89154-4003 USA; c Department of Materials Science and Engineering, Rensselaer Polytechnic Institute Troy New York 12180 USA

## Abstract

Novel fluorinated poly(aryl ether)s containing difluoromethylene (–CF_2_–) and tetrafluoroethylene (–CF_2_CF_2_–) segments were synthesized by nucleophilic aromatic substitution polymerization of bis(4-fluorophenyl)difluoromethane and 1,2-bis(4-fluorophenyl)-1,1,2,2-tetrafluoroethane. Properties of these partially fluorinated polymers were investigated in comparison with their carbonyl analogues, *i.e.* poly(aryl ether ketone)s and poly(aryl ether benzil)s, to study the effects of fluorinated moieties in the polymer backbone. These fluorinated polymers showed better solubility, higher thermal stability, and lower glass transition temperatures than those carbonyl containing polymers. Transparent, flexible and tough films were fabricated using either solution casting or compress molding, and they showed good UV transmittance, low dielectric constants and elastic moduli.

## Abstract 2

Novel fluorinated poly(aryl ether)s containing difluoromethylene (–CF_2_–) and tetrafluoroethylene (–CF_2_CF_2_–) segments were synthesized and their polymer properties with counterpart carbonyl [C(=O) and C(=O)C(=O)] containing polymers were studied.

## Graphical abstract

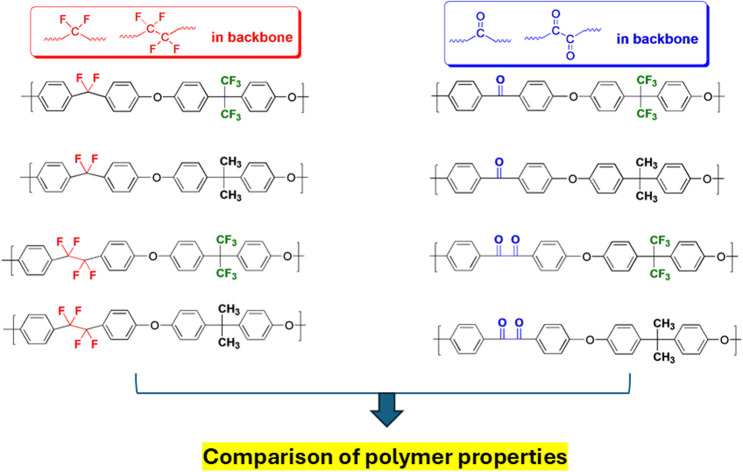

## Introduction

Poly(aryl ether)s are well known high-performance engineering thermoplastics with outstanding properties, including high thermal stability, chemical resistance, high modulus and toughness, and good insulating properties.^1^ They are generally synthesized by nucleophilic aromatic substitution polycondensations of bisphenoxides with aryl dihalides which are activated with electron-withdrawing groups, such as sulfones,^2^ ketones^3^ and phosphine oxides.^4^ In addition to those strong electron-withdrawing groups, other weakly electron-withdrawing functional groups, including heterocycles,^5^ thianthrene,^6^ azomethine,^7^ oxadiazole,^8^ triazole,^9^ benzimidazole,^10^ phenyl-quinoxaline,^11^ benzoxazole,^12^ amide,^13^ and fluoroalkyl groups,^14^ have also been employed to activate aryl halides.

Meanwhile, polymers containing fluorine or fluorinated groups possess many unique properties, such as high thermoxidative and chemical stability, a low dielectric constant, reduced optical loss and low moisture absorption.^15^ Among these, fluorinated poly(aryl ether)s are particularly noteworthy, finding wide application as films, coatings, and specialized membranes in advanced sectors such as electronics, optics, and aerospace.^16^

Based on their structural components, reported fluorinated poly(aryl ether)s are mainly classified into polymers containing: (1) a trifluoromethylated aromatic ring, (2) a hexafluoroisopropylidene (–C(CF_3_)_2_–) unit, and (3) hexafluorobenzene or decafluorobiphenyl segments.^17^ Delicate control of reaction conditions, especially temperature, is required for the polymerization with decafluorobiphenyl or hexafluorobezene due to the existence of multiple reactive sites that could cause crosslinking and gelation. As a result, it is difficult to achieve polymers with considerably high molecular weights. In addition, due to the limited availability of starting precursors, relatively few examples have been investigated with the fluorinated moieties in the polymer main chains. Labadie and coworkers reported the synthesis of highly fluorinated poly(perfluoroalkylene aryl ether)s.^18^ However, their work was constrained by the use of a perfluorohexyl group, which resulted in low glass transition temperatures (*T*_g_) for the resulting polymers, thereby preventing their application in high-temperature fields.

Our group previously reported an efficient direct fluorination of carbonyl groups of benzophenones and benzils with Deoxofluor. This method generated the corresponding products containing difluoromethylene or tetrafluoroethylene moieties in high yields.^19^ Using this method, aromatic dihalides containing difluoromethylene (–CF_2_–) and tetrafluoroethylene (–CF_2_CF_2_–) segments, which could serve as monomers for the synthesis of poly(aryl ether)s, were conveniently synthesized. In continuation of our interest in functional polymers with special applications in material science, we decided to demonstrate the feasibility of these fluorinated segments as activating groups for nucleophilic aromatic substitution polymerization and report herein the synthesis and properties of fluorinated poly(aryl ether)s. Although fluorinated materials have been documented with high optical transparency, high hydrophobicity and low dielectric constant, there is lack of direct comparison of these properties of fluorinated polymers with their hydrocarbon polymer counterparts. In this context, poly(aryl ether ketone)s and poly(aryl ether benzil)s, which contain –C(=O)– and –C(=O)C(=O)–, respectively, were synthesized and comparatively investigated with fluorinated poly(aryl ether)s to study the effect of the fluorinated moieties.

## Experimental section

### Materials and methods

All reactions were performed under a nitrogen atmosphere. ^1^H, ^13^C and ^19^F NMR spectra were obtained using a 400 MHz Varian NMR instrument at 400 MHz, 100 MHz and 376 MHz, respectively. NMP (extra dry with molecular sieves) was purchased from Acros and used as received. Toluene was dried by distillation and kept in the glove box. Size exclusion chromatography (SEC) analysis was conducted using a VISCOTEK chromatograph equipped with three visco-GEL I Series columns and tetra detector array at 40 °C. THF was the mobile phase, and the flow rate was set at 0.7 mL min^−1^. DSC and TGA measurement were conducted on a TA Q100 series under a nitrogen atmosphere. UV-visible transmittance was measured by Agilent Technologies, 8453 UV-Vis Spectrophotometer. The mechanical tests in tension were carried out using UTM, Instron 8516 at a constant crosshead speed of 25 mm min^−1^. Bis(4-fluorophenyl)difluoromethane and 1,2-bis(4-fluorophenyl)-1,1,2,2-tetrafluoroethane were synthesized according to our reported procedure.^19^ The static water contact angles of polymer films on glass plate were measured using a contact angle goniometer (Dataphysics OCA15) at room temperature. Ten measurements were performed for each film, and an average of these values is reported.

### Typical polymerization procedure of fluorinated poly(aryl ether)s

In a 100 mL round-bottom flask equipped with a magnetic stirring bar, a Dean–Stark apparatus fitted with a condenser and a nitrogen inlet, was charged with bis(4-fluorophenyl)difluoromethane (1.00 g, 4.16 mmol) and hexafluorobisphenol (1.40 g, 4.16 mmol), K_2_CO_3_ (1.73 g, 12.49 mmol, 3 equiv), NMP (20 mL) and toluene (20 mL). The reaction mixture was heated to reflux for 5 h as water was removed azeotropically by toluene. After toluene was removed from the Dean–Stark trap, the reaction mixture was heated to 180 °C and maintained stirring for 48 h. The reaction mixture was cooled to room temperature and added dropwise to a cold methanol (about 400 mL) with stirring. The fibrous solid was dissolved in chloroform, filtered through a short plug of silica gel and reprecipitated in methanol and afforded yellowish fibrous solid which was isolated *via* vacuum filtration and dried in vacuum to give 1.83 g of F2FBP (yield 82%). ^1^H NMR (CDCl_3_): *δ* 7.51 (d, *J* = 8.5 Hz, 4H), 7.38 (d, *J* = 8.5 Hz, 4H), 7.09 (d, *J* = 8.5 Hz, 4H), 7.01 (d, *J* = 9.0 Hz, 4H). ^19^F NMR (CDCl_3_): *δ* −64.5 (6F, C(C*F*_3_)_2_), −86.9 (2F, C*F*_2_). ^13^C NMR (CDCl_3_): *δ* 157.8, 157.5, 133.3 (t, ^2^*J*_C–F_ = 28.7 Hz), 132.1, 128.5, 128.1 (t, ^3^*J*_C–F_ = 5.6 Hz), 124.4 (quartet, ^1^*J*_C–F_ = 284.2 Hz), 120.6 (t, ^1^*J*_C–F_ = 240.4 Hz), 119.3, 118.4, 64.1 (quartet, ^2^*J*_C_–_F_ = 25.5 Hz).

Poly(aryl ether ketone)s were prepared using 4,4′-difluorobenzophenone in DMAc at 165 °C. Poly(aryl ether benzil)s were prepared using 4,4′-difluorobenzil in sulfolane at 160 °C by following a reported procedure.^20^

The fluorinated poly(aryl ether)s and poly(aryl ether ketone)s could be easily cast into flexible, transparent and tough films from NMP solution while chloroform was used for poly(aryl ether benzil)s. Compressing molding was employed for the film formation of samples for dielectric constant measurement, where 0.5 g polymer afforded film with a thickness range of 400–600 µm. Measurements of the dielectric constant were carried out in an Alpha Dielectric Analyzer from Novocontrol Technologies. All measurements were run from 10^−2^ to 10^6^ Hz at ambient temperatures of approximately 23 °C. Samples were used as is, with minor polishing in some cases to ensure good contact with the test cell electrodes.

## Results and discussion

### Polymer synthesis and characterization

**Scheme 1:**
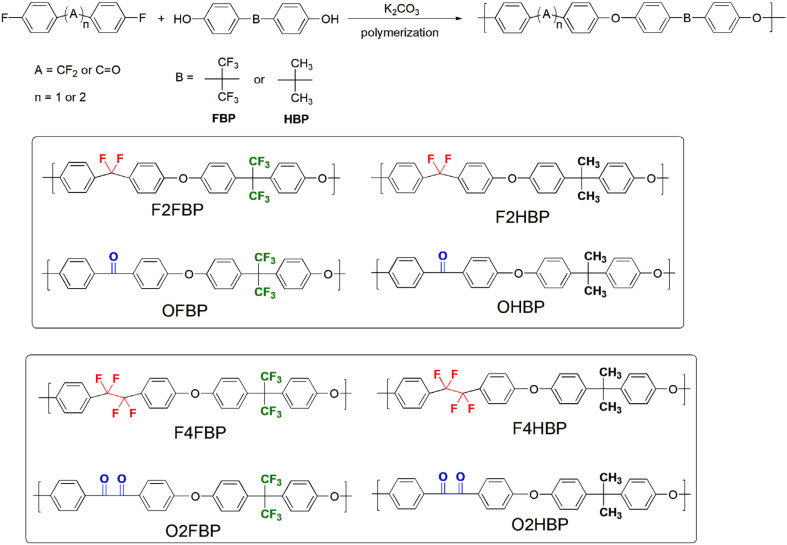
Synthesis of (fluorinated) poly(aryl ether)s.

As outlined in Scheme 1, polycondensations of bis(4-fluorophenyl)difluoromethane and 1,2-bis(4-fluorophenyl)-1,1,2,2-tetrafluoroethane with a stoichiometric amount of bisphenols (FBP and HBP of Scheme 1) were carried out in NMP as solvent in the presence of excess potassium carbonate. Polymerization yielded a dark, viscous solution, which was then precipitated into methanol to form fibrous polymers. Results showed that NMP was the best solvent to achieve a high degree of polymerization of the fluorinated poly(aryl ether)s; F2FBP, F2HBP, F4FBP, and F4HBP.

Poly(aryl ether ketone)s (OFBP and OHBP of Scheme 1) were prepared by the polycondensation of 4,4′-difluorobenzophenone with bisphenols in DMAc. The polymerization mixture became very viscous after stirring at 165 °C for 12 h, which was then precipitated into methanol to form a white fibrous solid. Poly(aryl ether benzil)s (O2FBP and O2HBP of Scheme 1) were prepared in a similar way using 4,4′-difluorobenzil as a monomer in polycondensation. For the polymerizations of benzil monomer, sulfolane proves to be a more effective medium than DMAc and NMP offering better solubility of the polymers.

**Fig. 1:**
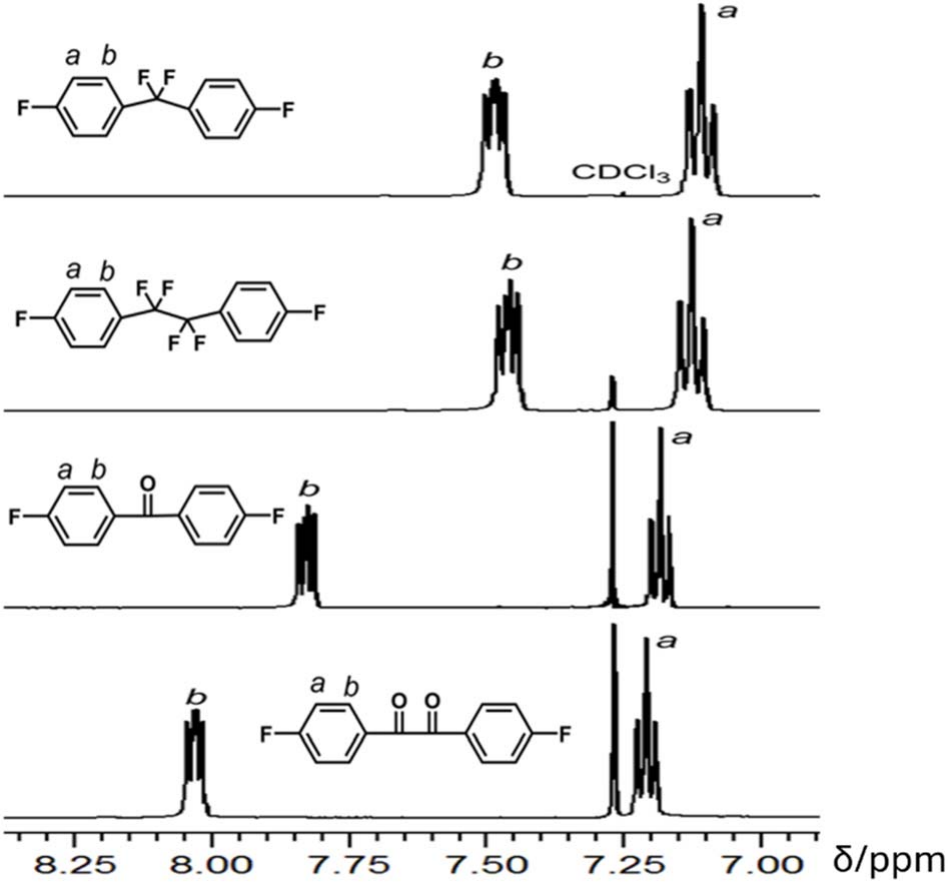
^1^H NMR spectra of aromatic difluoride monomers.

It is interesting to compare the potential reactivity of aromatic difluoride monomers towards the nucleophilic aromatic substitution reactions. The activating potential could be estimated by the chemical shift of the hydrogen *ortho* to the electron-withdrawing group:^20^ the more downfield shift generally indicates more enhanced reactivity toward nucleophilic aromatic substitution. As shown in Fig. 1, the *H*_b_ of 4,4′-difluorobenzil and 4,4′-difluorobenzophenone exhibit chemical shifts of 8.02 and 7.82 ppm, respectively. For comparison, the *H*_b_ of 1,2-bis(4-fluorophenyl)-1,1,2,2-tetrafluoroethane and bis(4-fluorophenyl)difluoromethane have more upfield shifts at 7.45 and 7.47 ppm, respectively. This result indicates that the tetrafluoroethylene and difluoromethylene units have lower electron-withdrawing inductive effects compared to their carbonyl analogous, and their aryl fluorides have lower reactivity towards phenoxides. It should be noted that the ^1^H NMR spectroscopic data provides reactivity estimation derived from inductive effect only. There could be other factors which can affect polymerization, such as stability of the Meisenheimer intermediate and the flexibility of polymer chain. For example, polymers prepared from 1,2-bis(4-fluorophenyl)-1,1,2,2-tetrafluoroethane generally have higher molecular weight that those from bis(4-fluorophenyl)difluoromethane despite of their *H*_b_ chemical shifts are similar in ^1^H NMR spectra, and this could be ascribed to the improved flexibility of the polymer chains.

**Fig. 2:**
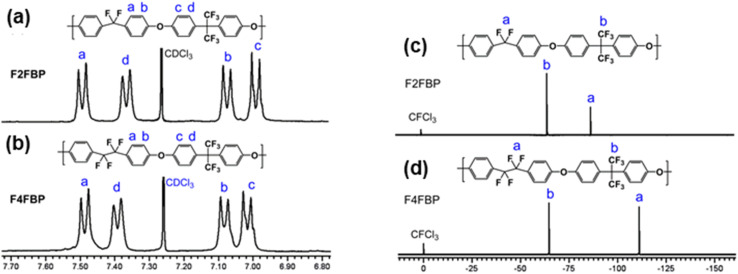
^1^H NMR spectra of (a) F2FBP and (b) F4FBP and ^19^F NMR spectra of (c) F2FBP and (d) F4FBP.

The fluorinated poly(aryl ether)s were characterized by NMR spectroscopy as shown in Fig. 2. Well resolved resonances of the polymer were obtained for both proton and fluorine NMR spectra and the integral ratios matched well with their polymer chemical structures. The difluoromethylene and tetrafluoroethylene groups showed characteristic chemical shifts at −86.9 and −111.0 ppm, respectively, in ^19^F NMR spectra. ^1^H and ^19^F NMR spectra of the polymers do not show any signals of the terminal –F and –OH groups, indicating a high degree of polymerization.

### Polymer properties

Molecular weights of poly(aryl ether)s were investigated *via* polystyrene-calibrated SEC employing tetrahydrofuran as the mobile phase. As illustrated in Table 1, most polymers have high molecular weights with dispersity (*M*_w_/*M*_n_) in the range of 1.9–3.2 which is typical for polycondensation polymers. Among aromatic difluoride monomers, 4,4′-difluorobenzophenone yielded the highest molecular weights with *M*_w_ up to 272 kg mol^−1^ for OHBP. As discussed before, the difluoromethylene moiety has lower inductive effect than the carbonyl counterpart and thus is less reactive towards phenoxides. For example, the molecular weights of F2HBP are much lower than those of OFBP and OHBP.

**Table 1:** Molecular weights of poly(aryl ether)s

Polymer	Solvent	Temp.^a^ (°C)	Time (h)	*M*_n_ b	*M*_w_ b	*M*_w_/*M*_n_
F2FBP	NMP	180	48	32 900	96 900	2.94
F2HBP	NMP	180	48	6000	14 500	2.41
OFBP	DMAc	165	12	98 100	189 400	1.93
OHBP	DMAc	165	12	143 200	272 200	1.90
F4FBP	NMP	180	48	69 100	219 900	3.19
F4HBP	NMP	180	48	31 100	70 100	2.25
O2FBP	Sulfolane	160	24	15 200	38 900	2.55
O2HBP	Sulfolane	160	24	23 000	48 900	2.13

a Polymerization reaction temperature.

b Measured by GPC at 40 °C with THF flow rate of 0.7 mL min^−1^.

Tetrafluoroethylene group containing polymers generally yielded higher molecular weights than difluoromethylene containing polymers, and this might be ascribed to the greater flexibility of polymer chains of the former. An interesting observation regarding the comparison of bisphenols HBP *vs.* FBP is that FBP tends to form higher molecular weights for –CF_2_– and –CF_2_CF_2_– containing polymers (*i.e.*, F2FBP and F4FBP), while HBP produces higher molecular weights for –C(=O)– and –C(=O)C(=O)– containing polymers (*i.e.*, OHBP and O2HBP). Although exact reason is unclear, it appears that fluorinated aromatic difluoride monomers like to react more with fluorinated FPB than non-fluorinated HBP and *vice versa*. Despite the strongest electron-withdrawing inductive effect of 4,4′-difluorobenzil, poly(aryl ether benzil)s (*i.e.*, O2FBP and O2HBP) tend to form lower molecular weights than poly(aryl ether ketone)s (*i.e.*, OFBP and OHBP) because of the rigid polymer backbone chains and low solubility of the formers.

### Polymer solubility

Good polymer solubility is essential for film formation and chemical modifications. The fluorinated poly(aryl ether)s exhibit good solubility behavior in common organic solvents, including NMP, DMAc, DMF, chloroform, toluene and THF because of the existence of flexible –O– linkage and the fluorinated segments in the backbone (Table S1). However, acetone and DMSO are not good solvents to dissolve these polymers. Both difluoromethylene and tetrafluoroethylene containing polymers exhibit similar solubility characteristics across a range of solvents.

### Optical properties

The high-molecular-weight fluorinated poly(aryl ether)s could be processed to transparent films conveniently by solution casting method with thickness ranging from 10 to 46 µm (Table 2). The replacement of C–H bonds with C–F bonds would increase the optical transparency of the polymeric material in the near-infrared (NIR) telecommunication region. These partially fluorinated polymer films exhibited good transparency, and the cut-off wavelength (*λ*_cutoff_) was in a range of 293 to 319 nm. The transparency at 800 nm ranges from 75 to 89%. The hexafluoroisopropylidene group (FBP) containing polymers showed higher transparency than their isopropylidene (HBP) containing counterparts.

**Table 2:** Thermal stability, water contact angle, and UV-Vis transmittance

Polymer	*T*_g_ (°C)^a^	*T*_d-5%_ (°C)^b^	*T*_d-10%_ (°C)	Water contact angle (°)	*λ*_cutoff_ (nm)^c^	UV-Vis trans. (%)^d^
F2FBP	153	490	510	96	295	87
F2HBP	109	439	482	—e	—e	—e
OFBP	172	521	533	96	345	90
OHBP	161	458	472	84	358	84
F4FBP	155	514	524	105	319	89
F4HBP	138	498	508	95	293	75
O2FBP	171	452	474	77	356	87
O2HBP	160	437	448	56	364	27

a *T*_g_ was determined by DSC at a heating rate of 10 °C min^−1^ under nitrogen (50 mL min^−1^), the values were reported from the second scan.

b Reported for 5% weight loss at a heating rate of 20 °C min^−1^ under air or nitrogen (flow rate 200 mL min^−1^).

c The specific wavelength below which a solvent or other component in a solution absorbs light so strongly that it interferes with the analysis of the sample.

d UV-Vis transmittance at 800 nm.

e Difficult to cast into film due to low molecular weights.

### Thermal and mechanical properties

Thermal behavior of polymers is affected by polymer structure. Glass transition temperatures of the fluorinated poly(aryl ether)s are consistently lower than their carbonyl counter parts (Table 2). Among polymers examined, FBP containing polymers showed higher *T*_g_ than HBP containing polymers. These *T*_g_ values are obviously higher than those of the polymers prepared from 1,6-bis(4-fluorophenyl)perfluorohexane which range from 40 to 95 °C.^18^ These fluorinated poly(aryl ether)s demonstrated excellent thermal stability with polymer decomposition temperature in 480–528 °C range (Table 2). Polymers containing a tetrafluoroethylene moiety are more thermally stable than those containing a difluoromethylene group. Hexafluoroisopropylidene group containing polymers exhibited better thermal stability compared to their hydrocarbon counterparts; for example, the 5% weight loss temperature of F4FBP is 16 °C higher than that of F4HBP.

The mechanical properties of the fluorinated poly(aryl ether)s varied substantially depending on the chemical structures and the curing temperatures (Table 3). The films had tensile strength of 35–48 MPa, elongations at break of 4.8–14.3%, and initial modulus of 1.5–1.8 GPa. These results indicate that the presence of fluorinated moieties improve solubility and optical transparency while negligibly affecting their mechanical properties.

**Table 3:** Mechanical properties of fluorinated poly(aryl ether)s

Polymer	Membrane thickness (µm)	Tensile modulus (GPa)	Tensile stress (MPa)	Elongation (%)
F2FBP	20	1.53	44.7	4.8
OHBP	53	1.81	35.2	2.1
F4FBP	25	1.67	48.0	3.3
O2HBP	32	1.59	47.0	14.3

### Surface properties and dielectric constant

**Fig. 3:**
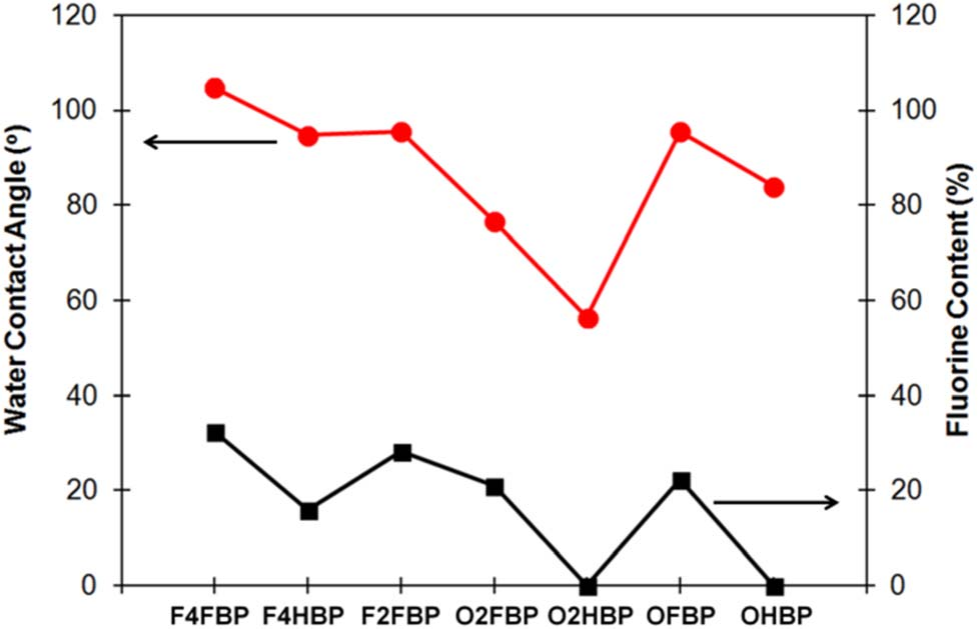
Water contact angle of fluorinated/non-fluorinated poly(aryl ether)s.

An important characteristic feature of fluorinated materials is their low surface energy, which directly influences the wetting and adhesion properties.^21^ Hydrophobicity of the films of fluorinated poly(aryl ether)s was investigated with water contact angle measurement in comparison with their non-fluorinated counterparts and the results were summarized in Table 2 and Fig. 3. Among them F4FBP showed the highest contact angle of 105° while O2HBP had the lowest contact angle of 56°. F4FBP's higher fluorine content and the low polarizability of C–F bonds lead to minimal interactions with water. To quantify the impact of fluorine contents on polymer's hydrophobicity, a plot of water contact angle *versus* fluorine content (wt%) is presented in Fig. 3. A clear correlation was observed; the trend of water contact angle strictly follows fluorine content regardless of the presence of the polar carbonyl group.

**Fig. 4:**
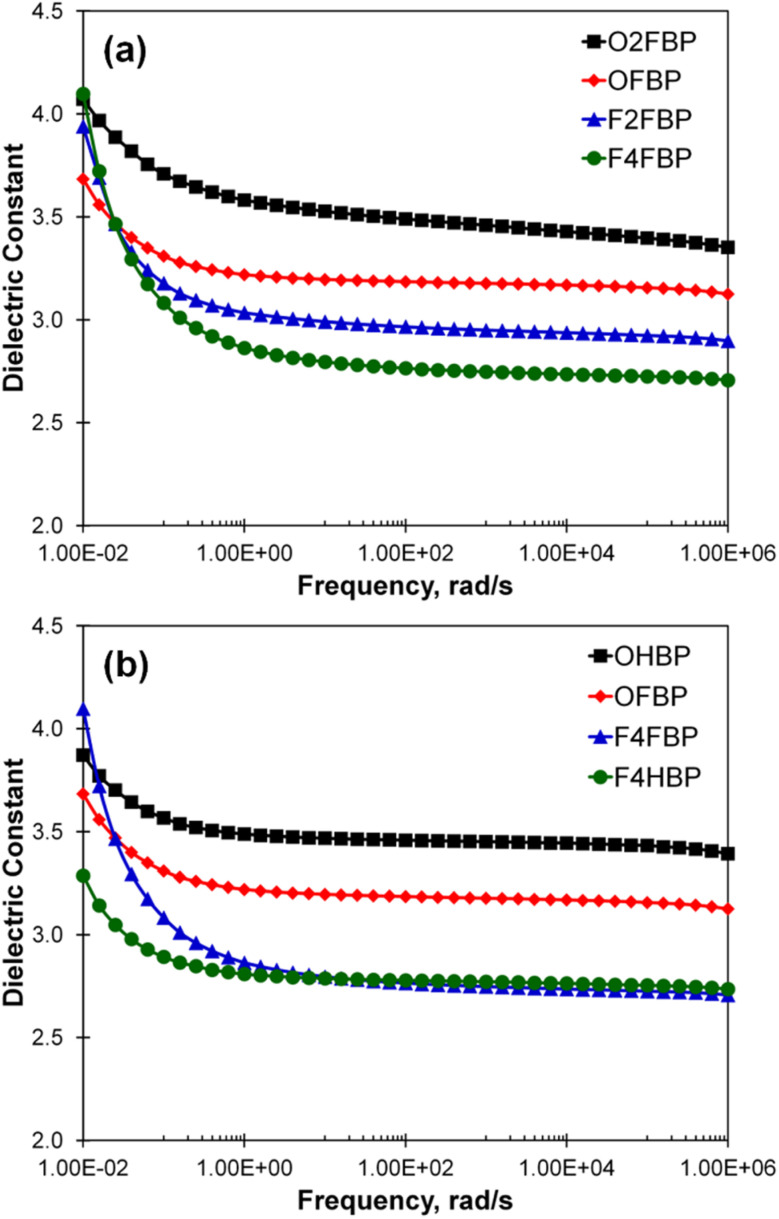
Dielectric constants data of fluorinate and non-fluorinated poly(aryl ether)s (please note that rad s^−1^ is for angular frequency (*ω*)) and Hz is for regular frequency (*f*) and both frequency units have the following relationship *f* = *ω*/2π.

Dielectric constants of the synthesized polymers are shown in Fig. 4 and Table S2. Among the polymers containing FBP, the dielectric constant systematically decreased in the order of O2FBP > OFBP > F2FBP > F4FBP (Fig. 4a). Similarly, FBP containing polymers have lower dielectric constants than HBP containing polymers. Because C=O bonds have higher polarizability than C–F bond, the dielectric constants of these polymers decrease with the increasing substitution of fluorine atoms. Furthermore, the higher free volume inherent to the fluorinated segments as compared to the carbonyl group, and the subsequent less efficient chain packing likely play a role in diminishing the dielectric constants.

## Conclusions

We have developed a series of novel fluorinated poly(aryl ether)s *via* nucleophilic aromatic substitution reaction by employing difluoromethylene and tetrafluoroethylene moieties as efficient activating groups of aromatic difluorides. Polymers containing a tetrafluoroethylene moiety generally have higher molecular weights and thermal stability. These partially fluorinated poly(aryl ether)s show good solubility, thermal stability, good optical transparency, high hydrophobicity and low dielectric constants. This study demonstrates that subtle modifications to the polymer backbone (*e.g.*, –CF_2_– *vs.* –C(=O)–, –CF_2_CF_2_– *vs.* –C(=O)C(=O)–, –C(CF_3_)_2_– *vs.* –C(CH_3_)_2_–) provide a flexible method for tailoring polymer properties.

## Author contributions

Y. C.: investigation, methodology, data curation, formal analysis, writing – original draft. A. A. I.: methodology, data curation, formal analysis. T. M. K.: methodology, data curation, formal analysis. M. R.: methodology, data curation, formal analysis. C. Y. R.: conceptualisation, supervision, L. S. S.: conceptualisation, supervision, C. B.: conceptualisation, supervision, funding acquisition, resources, validation, writing – review and editing.

## Conflicts of interest

There are no conflicts to declare.

## Supplementary Material

RA-015-D5RA08064A-s001

## Data Availability

The data supporting this article has been included as part of the supplementary information (SI). Supplementary information: ^1^H and ^19^F NMR spectra, solubility data, UV-vis spectra, DSC and TGA data. See DOI: https://doi.org/10.1039/d5ra08064a.
