# Supplementary material for: Fluorinated poly(aryl ether)s containing difluoromethylene and tetrafluoroethylene moieties
Source: RSC Adv. 2025 Dec 3;15(55):47630–6. doi: 10.1039/d5ra08064a (PMC12679576; doi:10.1039/d5ra08064a)
Supplement: RA-015-D5RA08064A-s001 [file RA-015-D5RA08064A-s001.pdf]

Supplementary Information (SI) for RSC Advances.
This journal is © The Royal Society of Chemistry 2025

Supplementary Information

Fluorinated Poly(aryl ether)s containing Difluoromethylene and Tetrafluoroethylene Moieties

*Ying Chang,[1,2] Adi Avi-Izak,[2] Timothy M. Krentz,[3] Matthew Ravalli, [1] Chang Y. Ryu, [1] and Chulsung Bae[1,2]**

[1]Department of Chemistry and Chemical Biology, New York State Center for Polymer Synthesis, Rensselaer Polytechnic Institute, Troy, New York 12180

[2]Department of Chemistry, University of Nevada Las Vegas, 4505 Maryland Parkway, Box 454003, Las Vegas, Nevada 89154-4003

[3]Department of Materials Science and Engineering and Rensselaer Nanotechnology Center, Rensselaer Polytechnic Institute, Rensselaer Polytechnic Institute, Troy, New York 12180

General Materials and Methods:

^{1}H, ^{19}F and ^{13}C NMR spectra were obtained using a 400 MHz, 376MHz and 100MHz Varian NMR spectrometer at room temperature and chemical shifts were referenced to TMS (^{1}H) and $CFCl_3$ (^{19}F). GC/MS analysis was conducted using a Shimadzu ----- equipped with a 30 m × 0.25 mm SPB-5 GC column and an EI ionization MS detector. DSC and TGA measurement were conducted on a TA Q100 series under a nitrogen atmosphere.

F2FBP ^{1}H NMR ($CDCl_3$): δ 7.51 (d, J = 8.5 Hz, 4H), 7.38 (d, J = 8.5 Hz, 4H), 7.09 (d, J = 8.5 Hz, 4H), 7.01 (d, J = 9.0 Hz, 4H). ^{19}F NMR ($CDCl_3$): δ –64.5 (6F, $C(CF_3)_2$), –86.9 (2F, CF_2). ^{13}C NMR ($CDCl_3$): δ 157.8, 157.5, 133.3 (t, $^{2}J_{C\text{-}F}$ = 28.7 Hz), 132.1, 128.5, 128.1 (t, $^{3}J_{C\text{-}F}$ = 5.6 Hz), 124.4 (quartet, $^{1}J_{C\text{-}F}$ = 284.2 Hz), 120.6 (t, $^{1}J_{C\text{-}F}$ = 240.4 Hz), 119.3, 118.4, 64.1 (quartet, $^{2}J_{C\text{-}F}$ = 25.5 Hz).

F2HBP ^{1}H NMR ($CDCl_3$): δ 7.42 (d, J = 8.4 Hz, 4H), 7.20 (d, J = 8.4 Hz, 4H), 6.98 (d, J = 8.4 Hz, 4H), 6.92 (d, J = 8.8 Hz, 4H), 1.70 (s, 6H). ^{19}F NMR ($CDCl_3$): δ –86.2 (2F, CF_2). ^{13}C NMR ($CDCl_3$): δ 159.2, 154.3,

146.5, 132.3 (t, $^{2}J_{C\text{-}F}$ = 28.7 Hz), 128.4, 127.8 (t, $^{3}J_{C\text{-}F}$ = 5.3 Hz), 120. 9 (t, $^{1}J_{C\text{-}F}$ = 239.7 Hz), 119.3, 118.1, 42.5, 31.3.

F4FBP ^{1}H NMR ($CDCl_3$): δ 7.49 (d, *J* = 8.4 Hz, 4H), 7.39 (d, *J* = 8.4 Hz, 4H), 7.08 (d, *J* = 8.4 Hz, 4H), 7.01 (d, *J* = 9.2 Hz, 4H). ^{19}F NMR ($CDCl_3$): δ –64.5 (6F, $C(CF_3)_2$), –111.1 (4F, CF_2CF_2). ^{13}C NMR ($CDCl_3$): δ 158.8, 157.2, 132.2, 129.2 (t, $^{3}J_{C\text{-}F}$ = 3.7 Hz), 128.7, 126.3 (t, $^{2}J_{C\text{-}F}$ = 25.7 Hz), 124.4 (quartet, J = 285.0 Hz), 118.9, 118.7, 116.7 (tt, $^{1}J_{C\text{-}F}$ = 251.5 Hz, $^{2}J_{C\text{-}F}$ = 37.2 Hz), 64.1 (m).

F4HBP ^{1}H NMR ($CDCl_3$): δ 7.40 (d, *J* = 8.4 Hz, 4H), 7.23 (d, *J* = 8.4 Hz, 4H), 6.99 (d, *J* = 8.8 Hz, 4H), 6.95 (d, *J* = 7.6 Hz, 4H), 1.69 (s, 6H). ^{19}F NMR ($CDCl_3$): δ –111.1 (4F, CF_2CF_2). ^{13}C NMR ($CDCl_3$): δ 160.2, 154.0, 146.7, 128.9 (t, $^{3}J_{C\text{-}F}$ = 3.8 Hz), 128.5, 125.3 (t, $^{2}J_{C\text{-}F}$ = 22.3 Hz), 119.5, 117.7, 116.9 (tt, $^{1}J_{C\text{-}F}$ = 251.5 Hz, $^{2}J_{C\text{-}F}$ = 36.5 Hz), 42.5, 31.2.

OFBP ^{1}H NMR ($CDCl_3$): δ 7.84 (d, J = 8.5 Hz, 4H), 7.42 (d, J = 8.0 Hz, 4H), 7.11 (d, J = 8.5 Hz, 4H), 7.07 (d, J = 8.5 Hz, 4H). ^{19}F NMR ($CDCl_3$): δ –64.5 (6F, $C(CF_3)_2$). ^{13}C NMR ($CDCl_3$): δ. 194.2, 160.1, 156.6, 133.0, 132.4, 131.2, 128.8, 124.2 (quartet, $^{1}J_{C\text{-}F}$ = 287.2 Hz), 118.9, 118.3, 63.8 (m).

OHBP ^{1}H NMR ($CDCl_3$): δ 7.79 (d, J = 8.5 Hz, 4H), 7.42 (d, J = 8.0 Hz, 4H), 7.28 (d, J = 8.5 Hz, 4H), 7.04 (d, J = 9.0 Hz, 4H), 7.01 (d, J = 9.0 Hz, 4H). ^{13}C NMR ($CDCl_3$): δ 195.0, 161.5, 153.4, 146.8, 132.3, 132.2, 128.4, 119.6, 117.2, 42,4, 31.1.

O2FBP ^{1}H NMR ($CDCl_3$): δ 7.99 (d, J = 9.0 Hz, 4H), 7.43 (d, J = 8.5 Hz, 4H), 7.10 (d, J = 8.0 Hz, 4H), 7.07 (d, J = 8.5 Hz, 4H). ^{19}F NMR ($CDCl_3$): δ –64.5 (6F, $C(CF_3)_2$). ^{13}C NMR ($CDCl_3$): δ 192.8, 163.2, 155.9, 132.5, 132.1, 129.4, 128.3, 124.1 (quartet, $^{1}J_{C\text{-}F}$ = 287.2 Hz), 119.5, 118.4, 63.9 (m).

O2HBP ^{1}H NMR ($CDCl_3$): δ 7.93, 7.27, 7.28, 7.01, 6.99, 1.71 (6H). ^{13}C NMR ($CDCl_3$): δ 193.5, 163.7, 152.7, 147.3, 132.4, 128.5, 127.5, 120.0, 117.4, 42.5, 31.0.

Table S1. Solubility data of (fluorinated) poly(aryl ether)s. [a]

Polymer	NMP	DMAc	DMF	DMSO	THF	acetone	$CHCl_3$	toluene
**F2FBP**	+	+	+	o	+	+	+	+
**F2HBP**	+	+	+	o	+	–	+	+
**OFBP**	+	+	±	o	+	–	+	+
**OHBP**	+	+	–	–	+	–	+	–
**F4FBP**	+	+	+	±	+	+	+	+
**F4HBP**	+	+	+	o	+	–	+	+
**O2FBP**	+	+	+	+	+	±	+	+
**O2HBP**	+	+	+	±	+	–	+	–

[a] (+) soluble at room temperature; (±) soluble on heating, remaining clear cooled to room temperature; (o) soluble on heating, became cloudy on cooling to room temperature; (–) not completely soluble under refluxing.

Table S2. Thermal stability, water contact angle, and UV-vis transmittance.

Polymer	T_g (°C)[a]	$T_{d\text{-}5\%}$ (°C)[b]	$T_{d\text{-}10\%}$ (°C)	Water contact angle (°)	λ_{cutoff} (nm)	UV-vis trans. (%)[c]	Thickness (μm)
**F2FBP**	153	490	510	96	295	87	20
**F2HBP**	109	439	482	--[d]	--[d]	--[d]	--[d]
**OFBP**	172	521	533	96	345	90	61
**OHBP**	161	458	472	84	358	84	53
**F4FBP**	155	514	524	105	319	89	25
**F4HBP**	138	498	508	95	293	75	--[e]
**O2FBP**	171	452	474	77	356	87	--[e]
**O2HBP**	160	437	448	56	364	27	32

[a] T_g was determined by DSC at a heating rate of 10 °C min^{-1} under nitrogen (50 mL min^{-1}), the values were reported from the second scan. [b] Reported for 5% weight loss at a heating rate of 20 °C min^{-1} under air or nitrogen (flow rate 200 mL min^{-1}).[c] UV-transmittance at 800 nm. [d] Difficult to cast into film due to low molecular weights. [e] No information available.

Tabel S3. Dielectric constants data of fluorinate and non-fluorinated poly(aryl ether)s.

Polymer	100Hz	10 kHz	1MHz
**F2FBP**	2.97	2.94	2.90
**OFBP**	3.18	3.17	3.12
**OHBP**	3.46	3.45	3.39
**F4FBP**	2.76	2.74	2.71
**F4HBP**	2.78	2.76	2.74
**O2FBP**	3.49	3.43	3.35

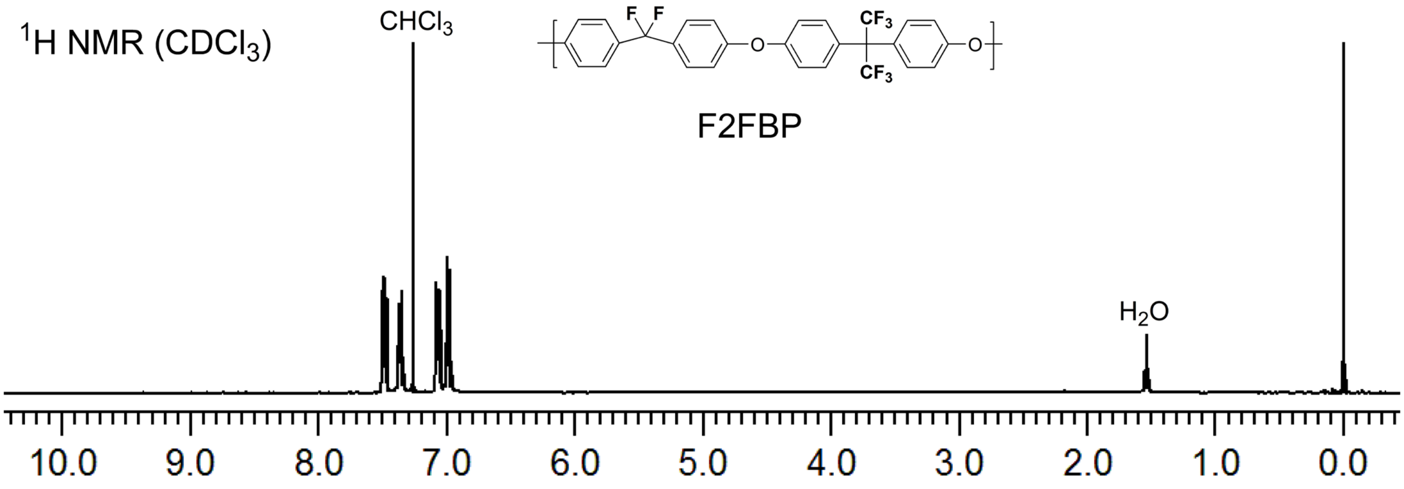

Figure S1. ^{1}H NMR spectrum of F2FBP.

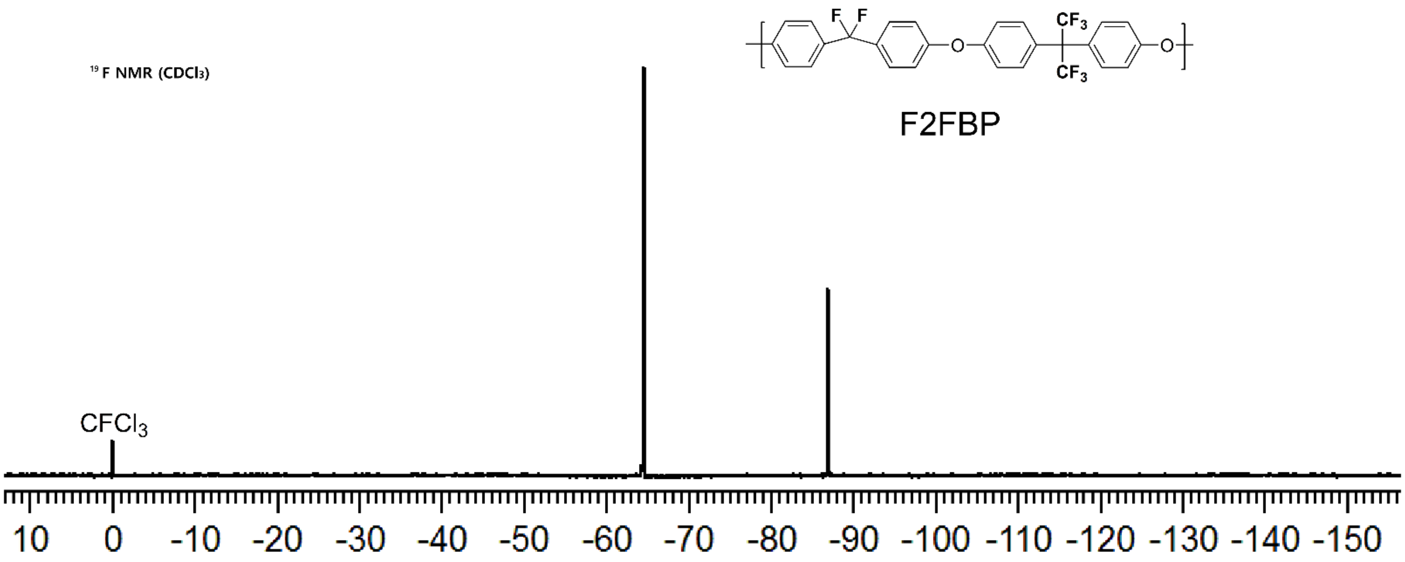

Figure S2. ^{19}F NMR spectrum of F2FBP.

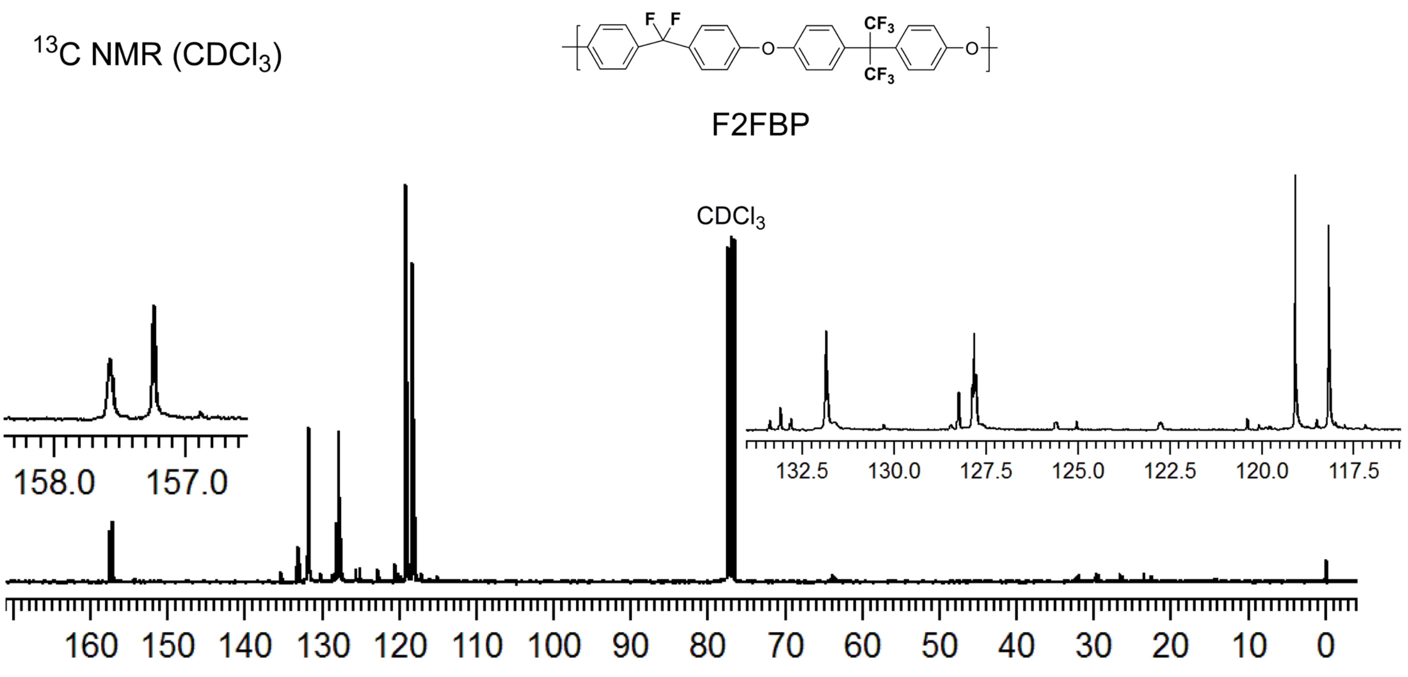

Figure S3. ^{13}C NMR spectrum of F2FBP.

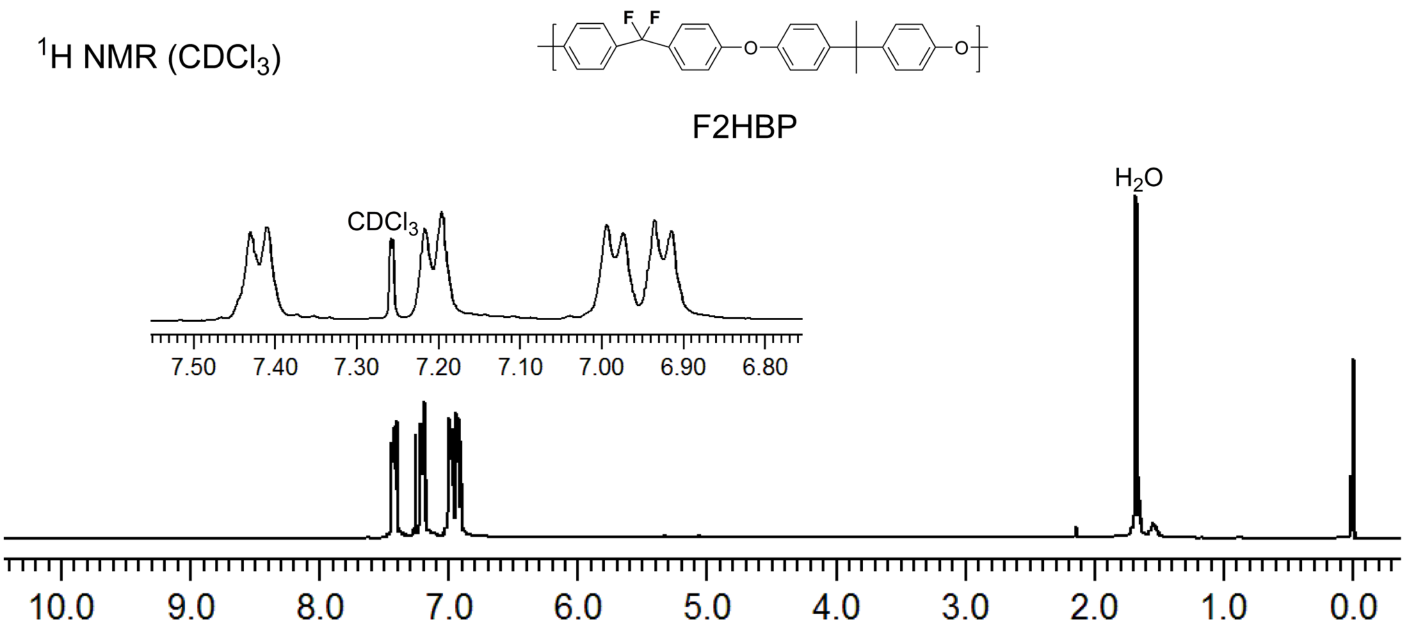

Figure S4. ^{1}H NMR spectrum of F2HBP.

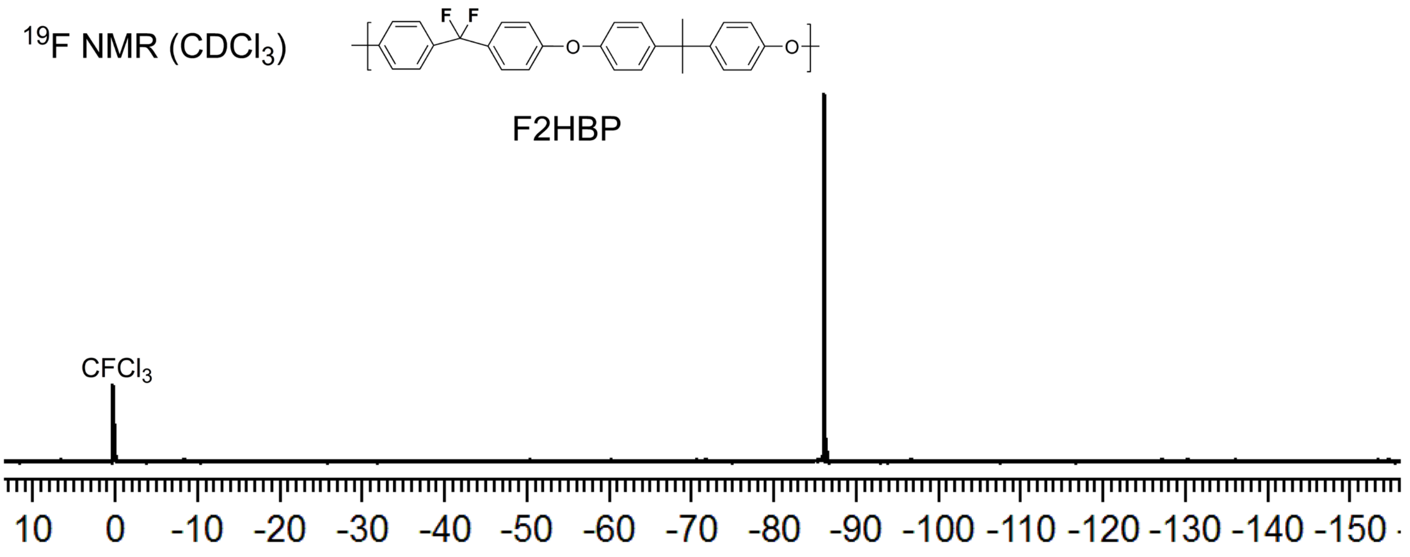

Figure S5. ^{19}F NMR spectrum of F2HBP.

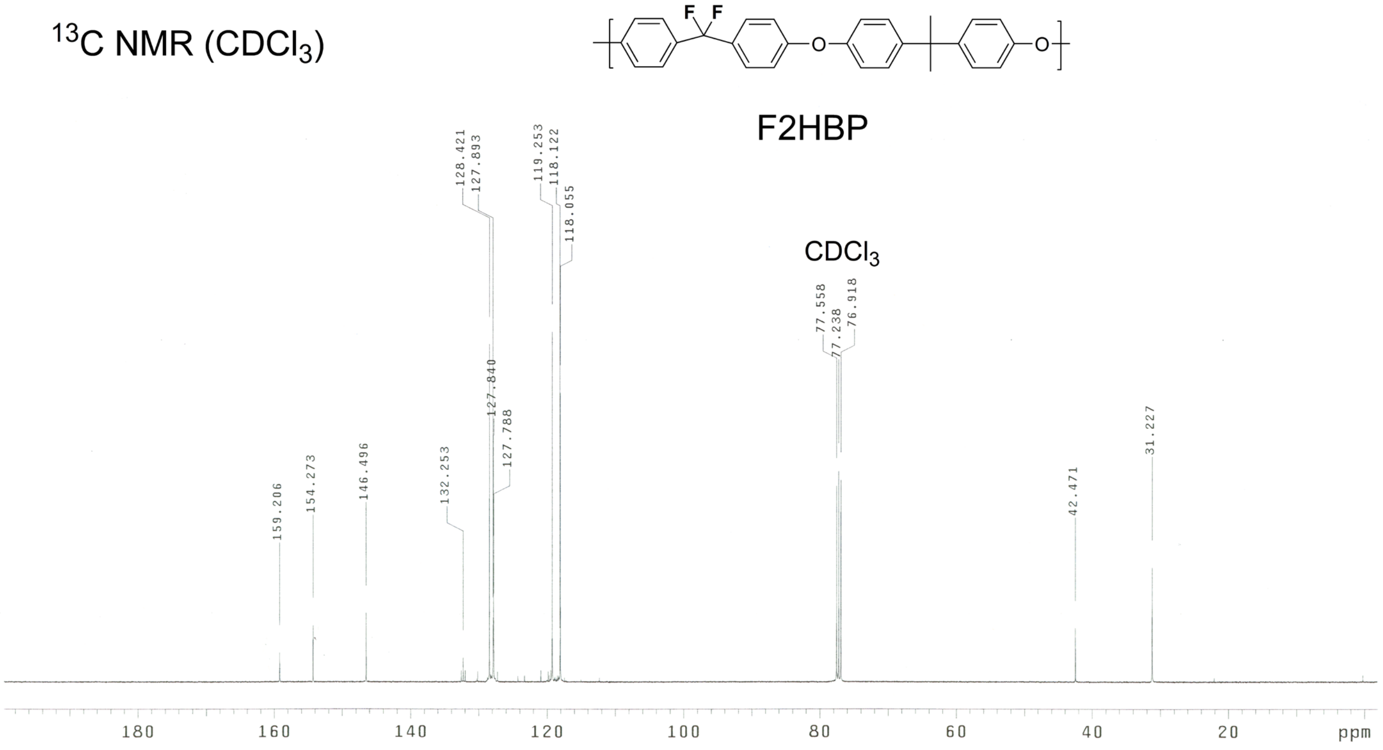

Figure S6. ^{13}C NMR spectrum of F2HBP.

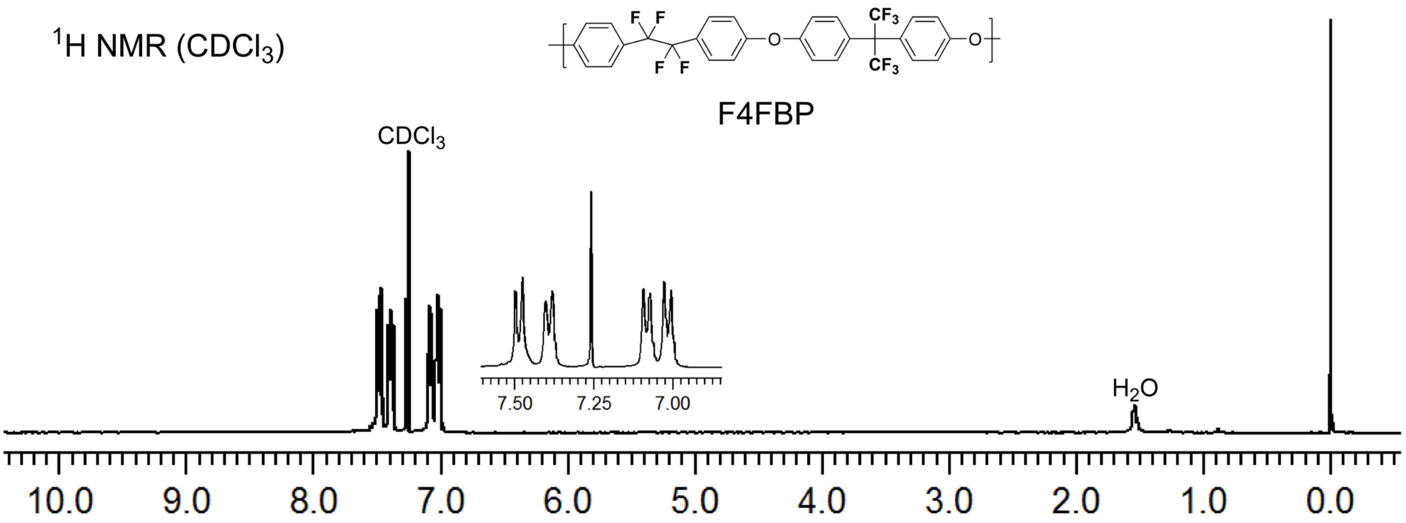

Figure S7. ^{1}H NMR spectrum of F4FBP.

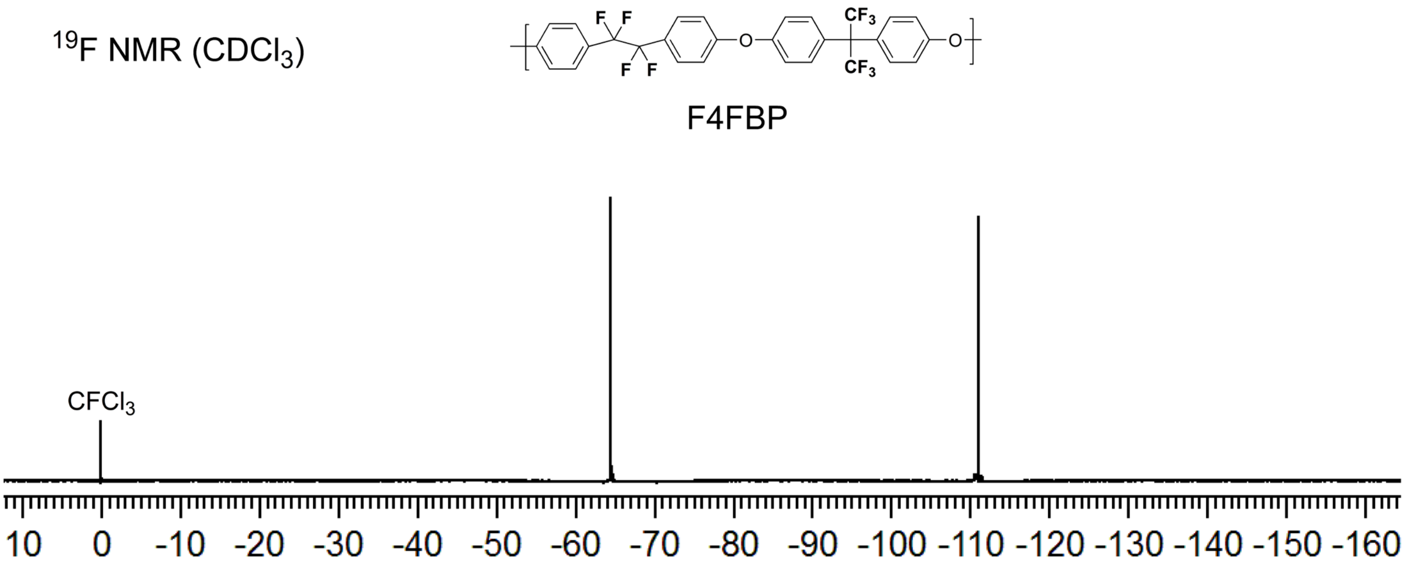

Figure S8. ^{19}F NMR spectrum of F4FBP.

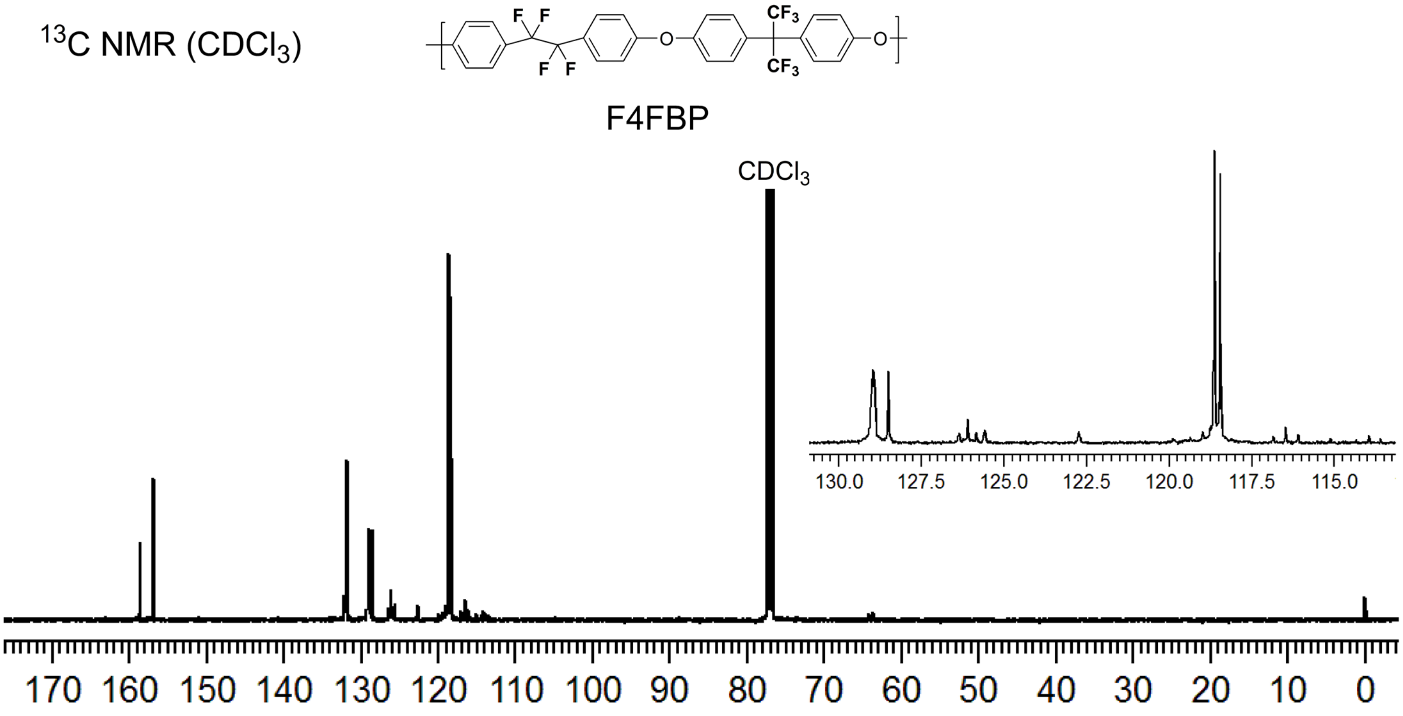

Figure S9. ^{13}C NMR spectrum of F4FBP.

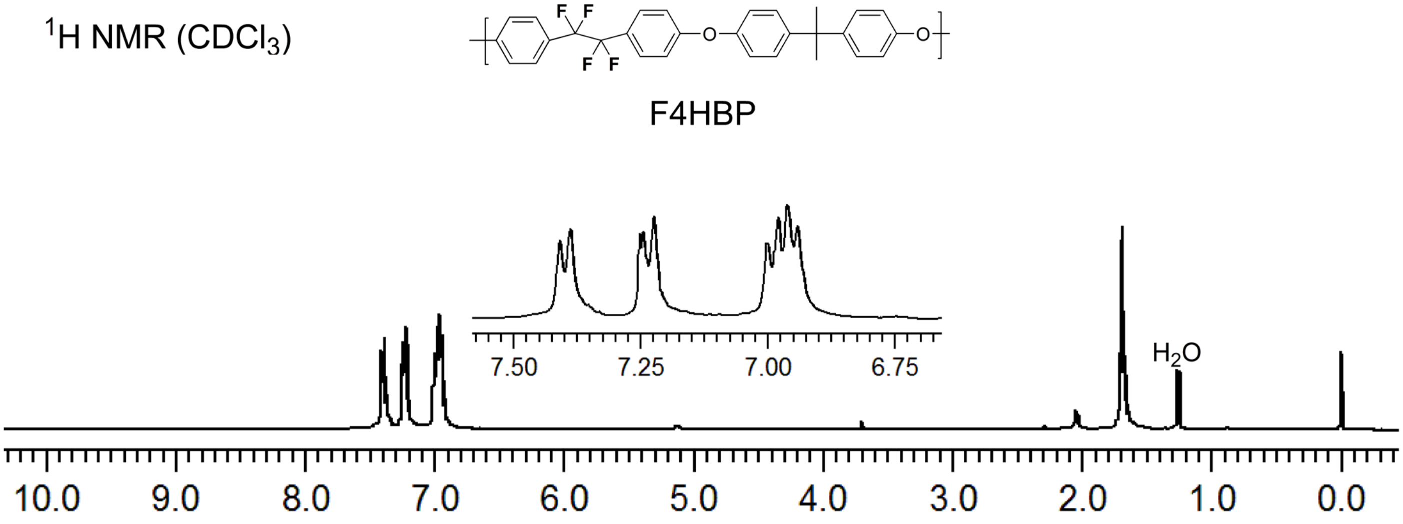

Figure S10. ^{1}H NMR spectrum of F4HBP.

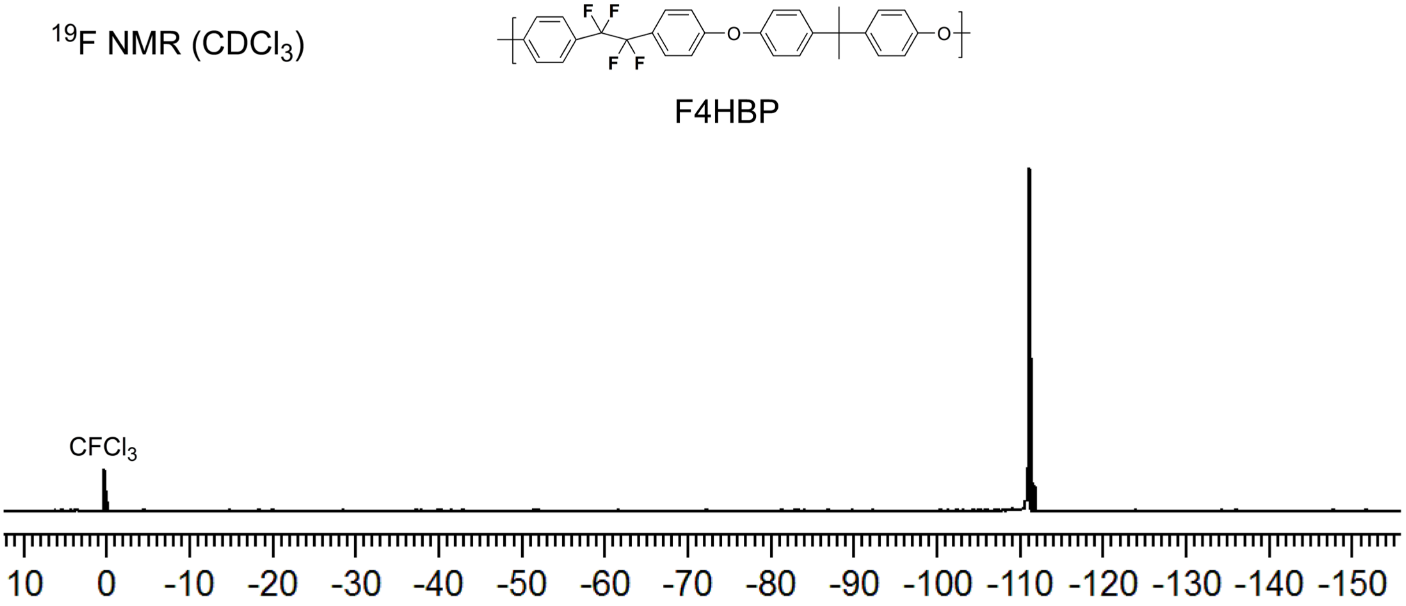

Figure S11. ^{19}F NMR spectrum of F4HBP.

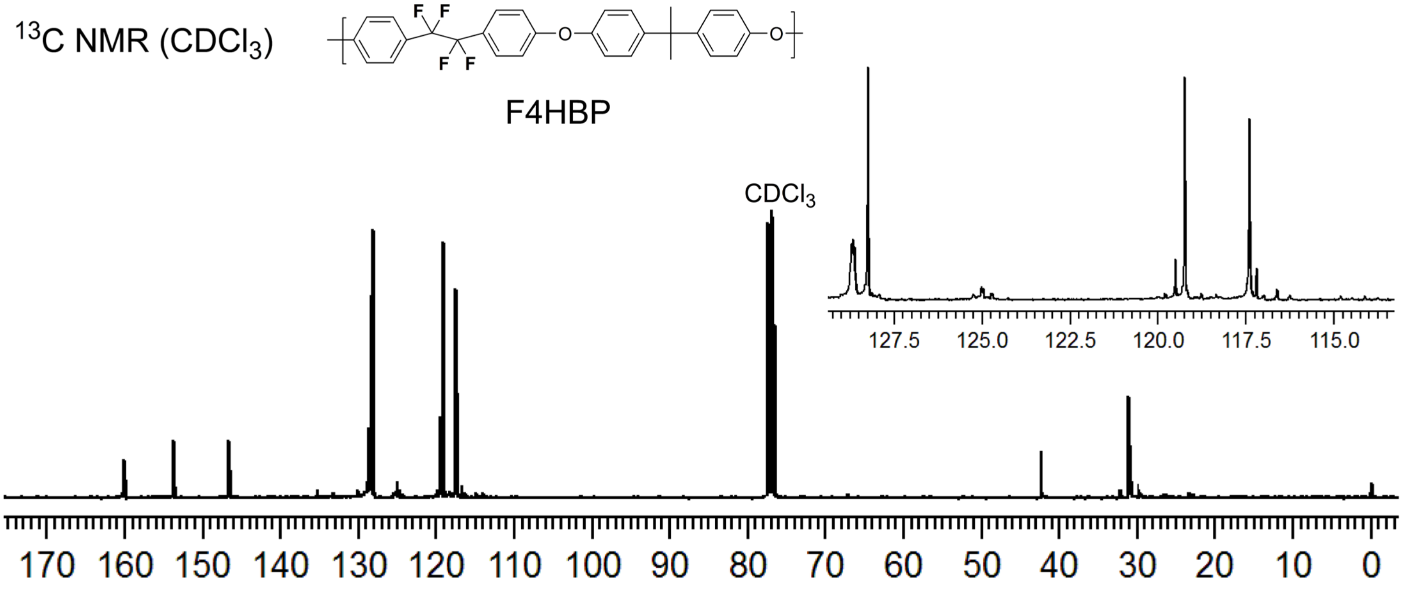

Figure S12. ^{13}C NMR spectrum of F4HBP.

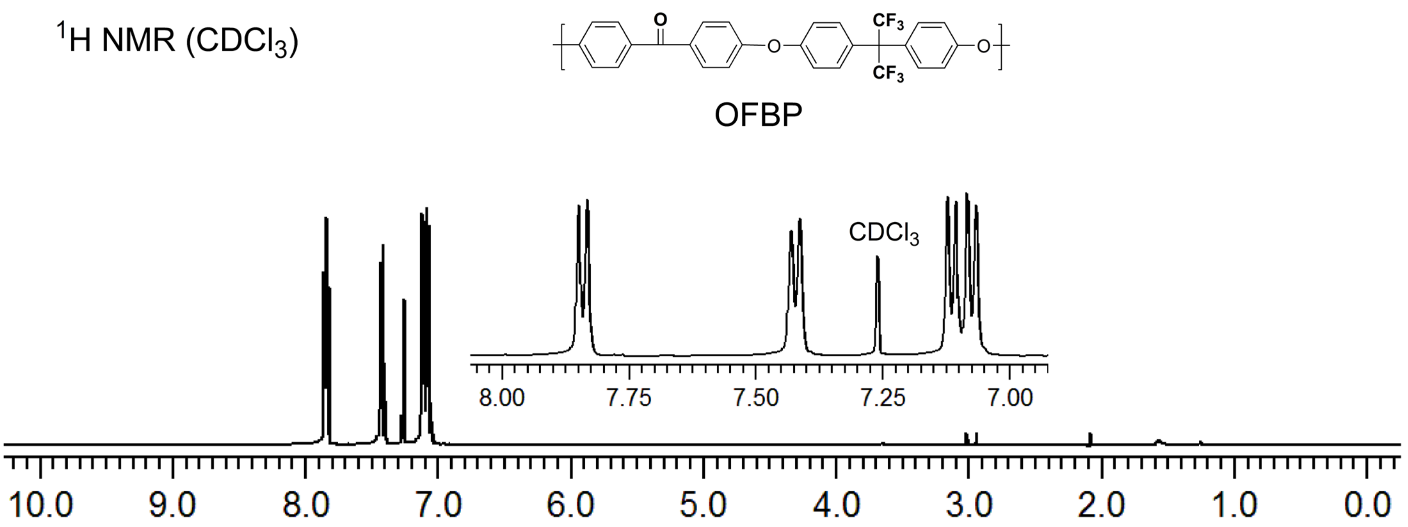

Figure S13. ^{1}H NMR spectrum of OFBP.

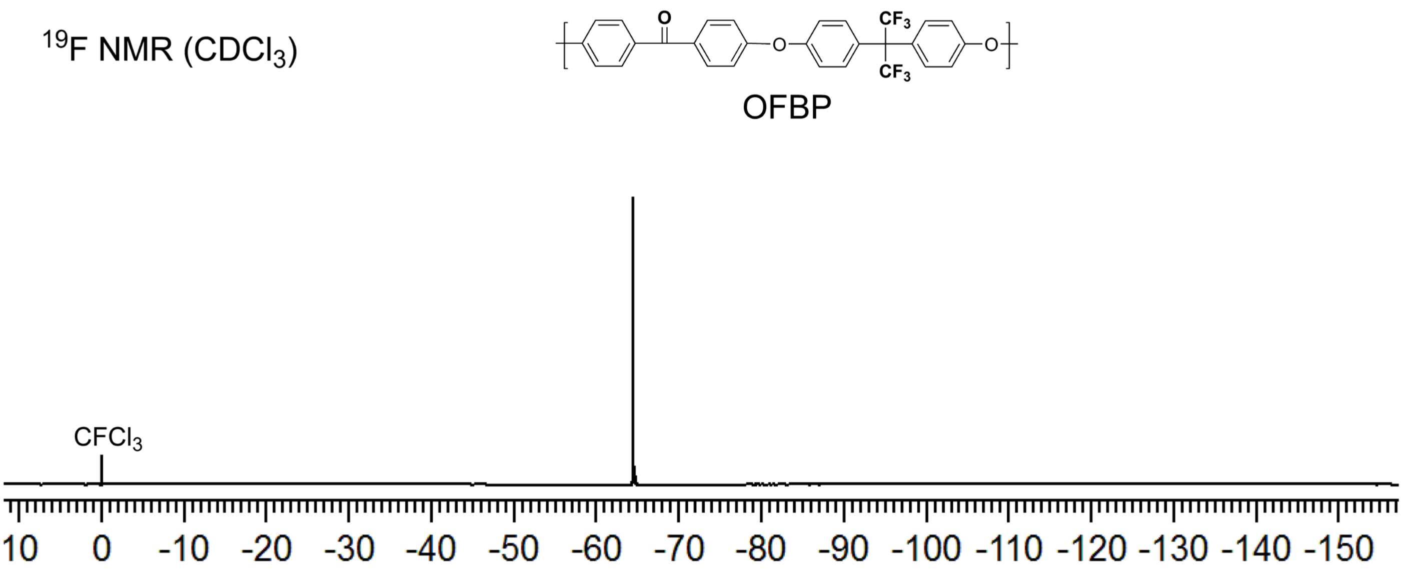

Figure S14. ^{19}F NMR spectrum of OFBP.

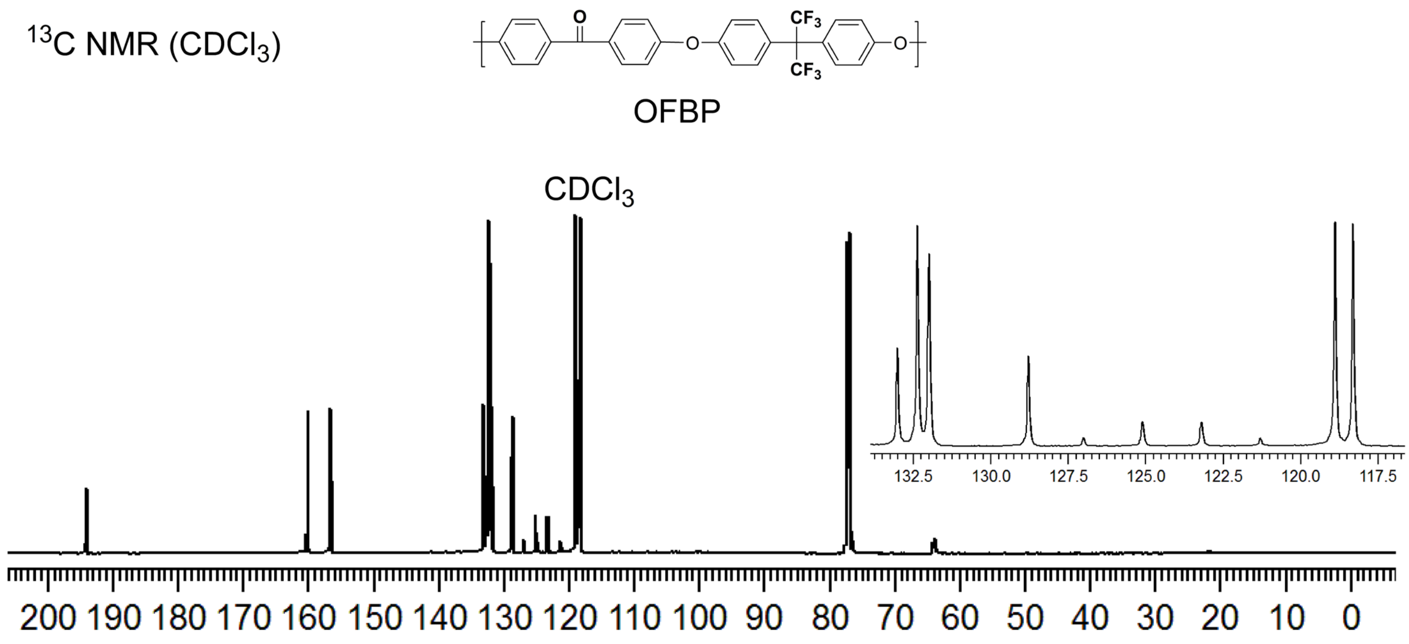

Figure S15. ^{13}C NMR spectrum of OFBP.

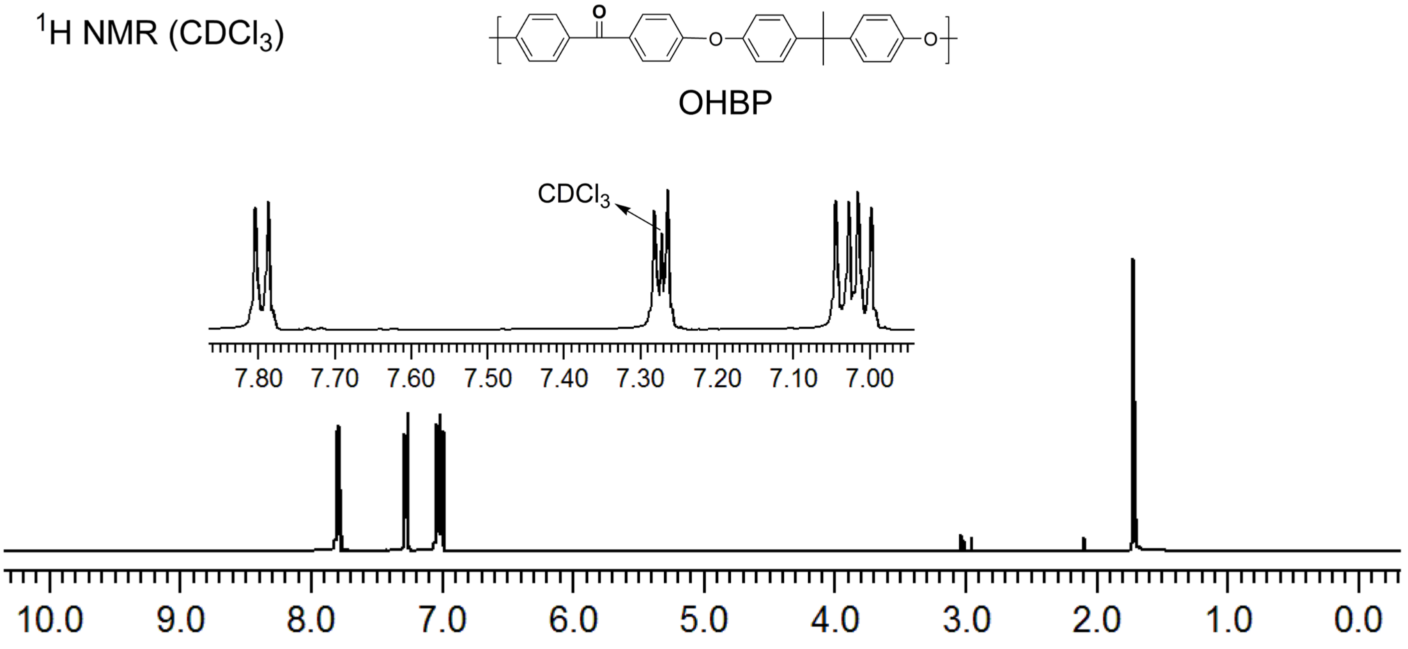

Figure S16. ^{1}H NMR spectrum of OHBP.

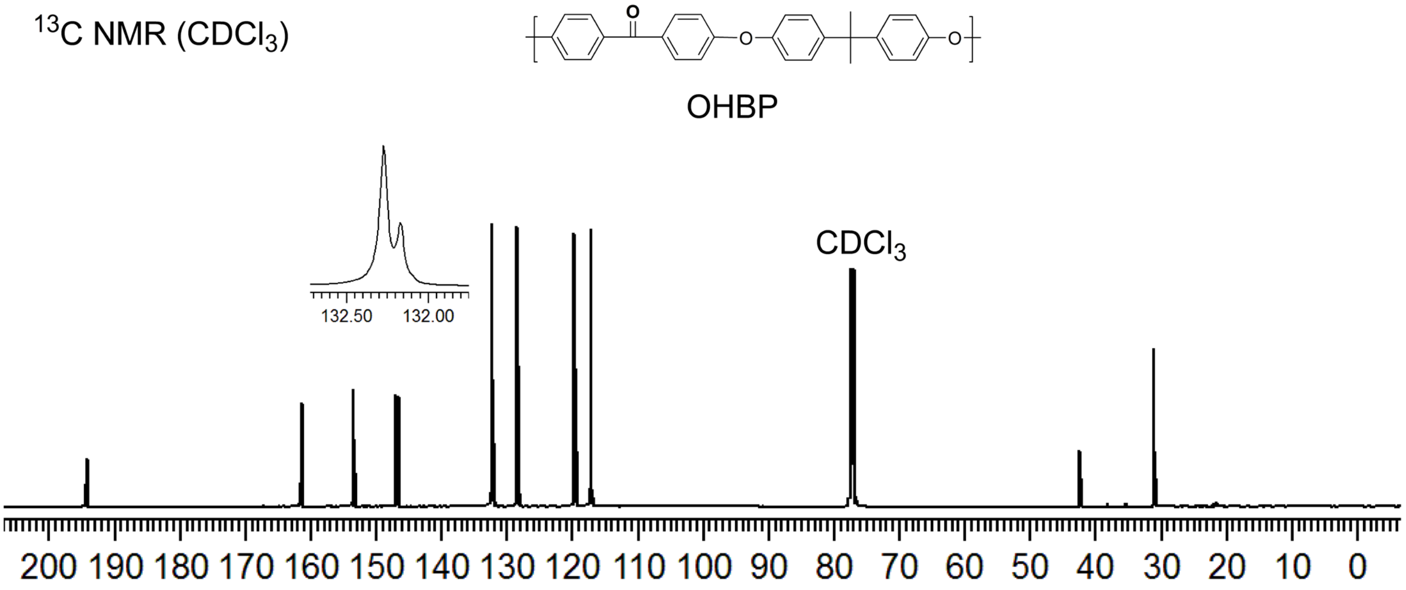

Figure S17. ^{13}C NMR spectrum of OHBP.

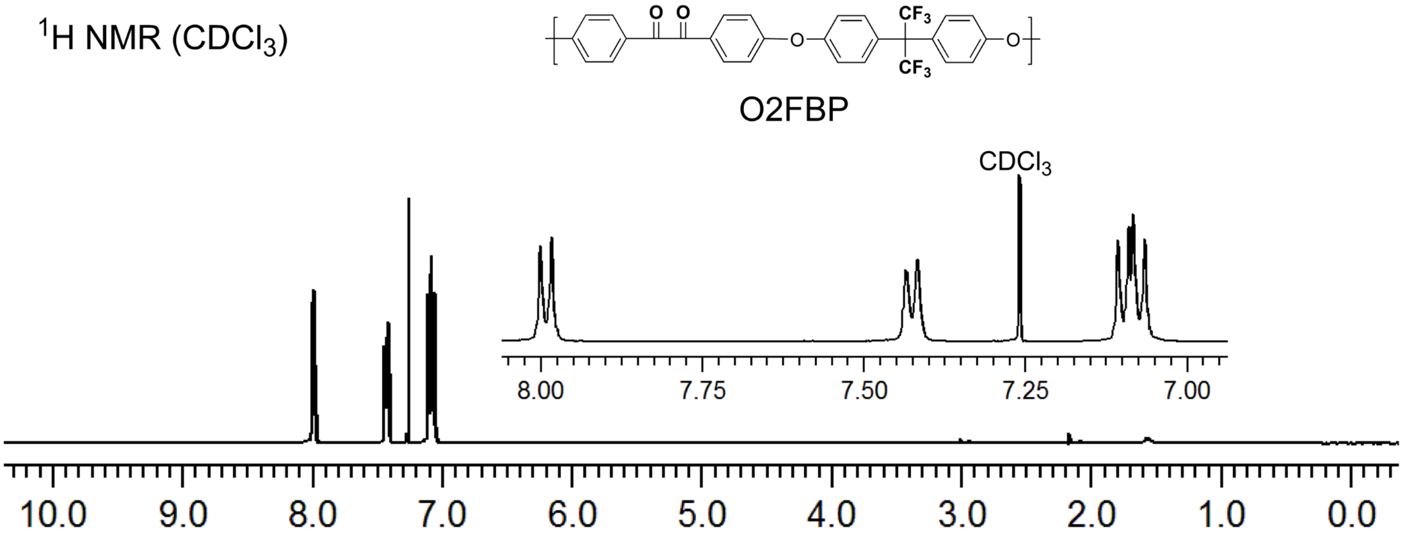

Figure S18. ^{1}H NMR spectrum of O2FBP.

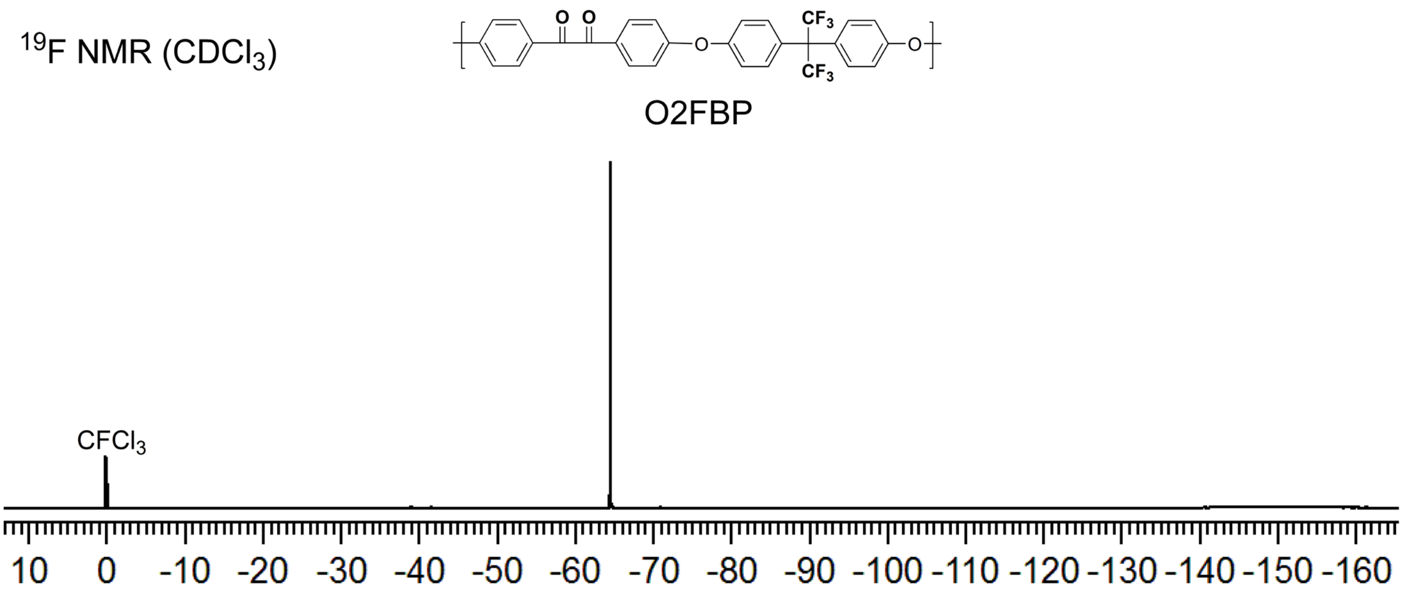

Figure S19. ^{19}F NMR spectrum of O2FBP.

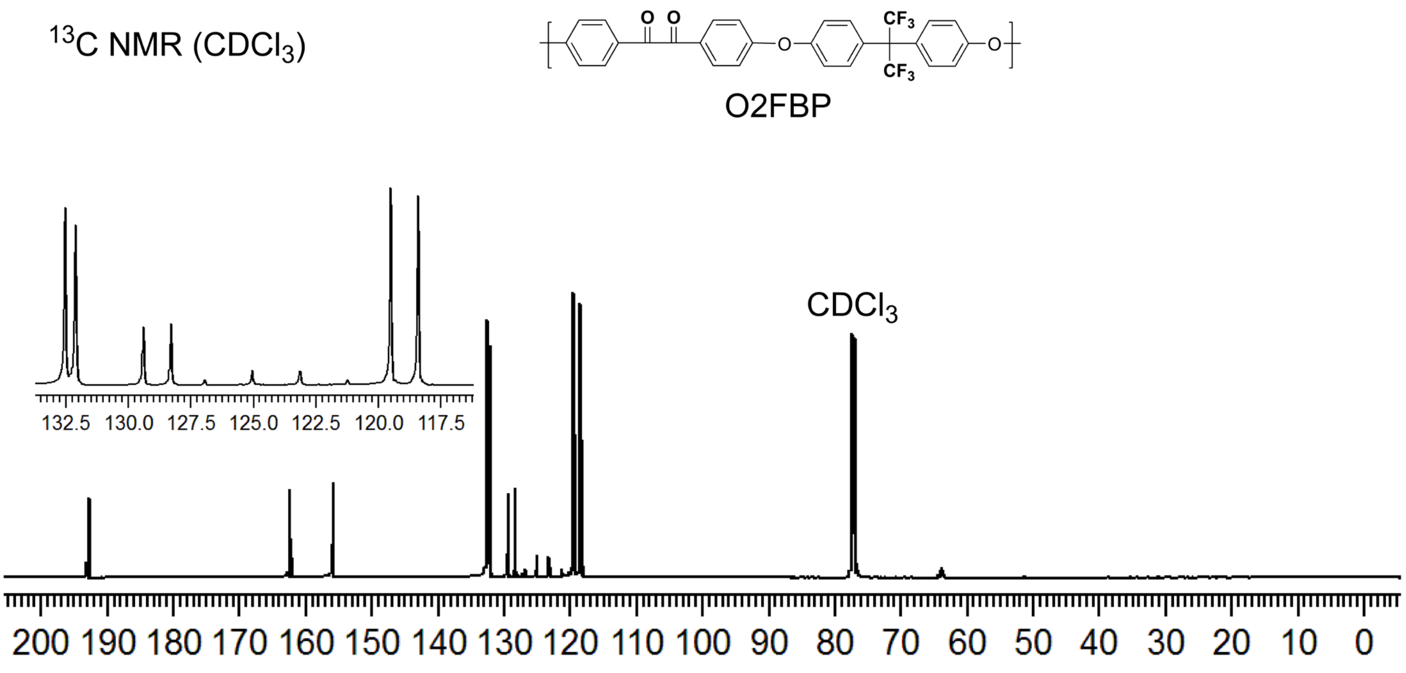

Figure S20. ^{13}C NMR spectrum of O2FBP.

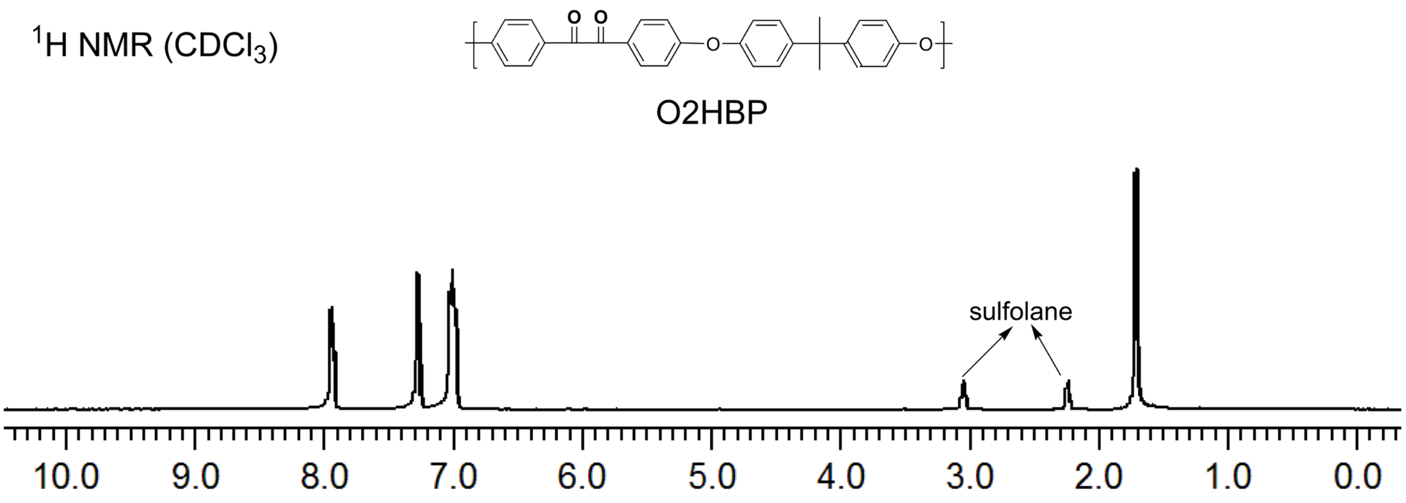

Figure S21. ^{1}H NMR spectrum of O2HBP.

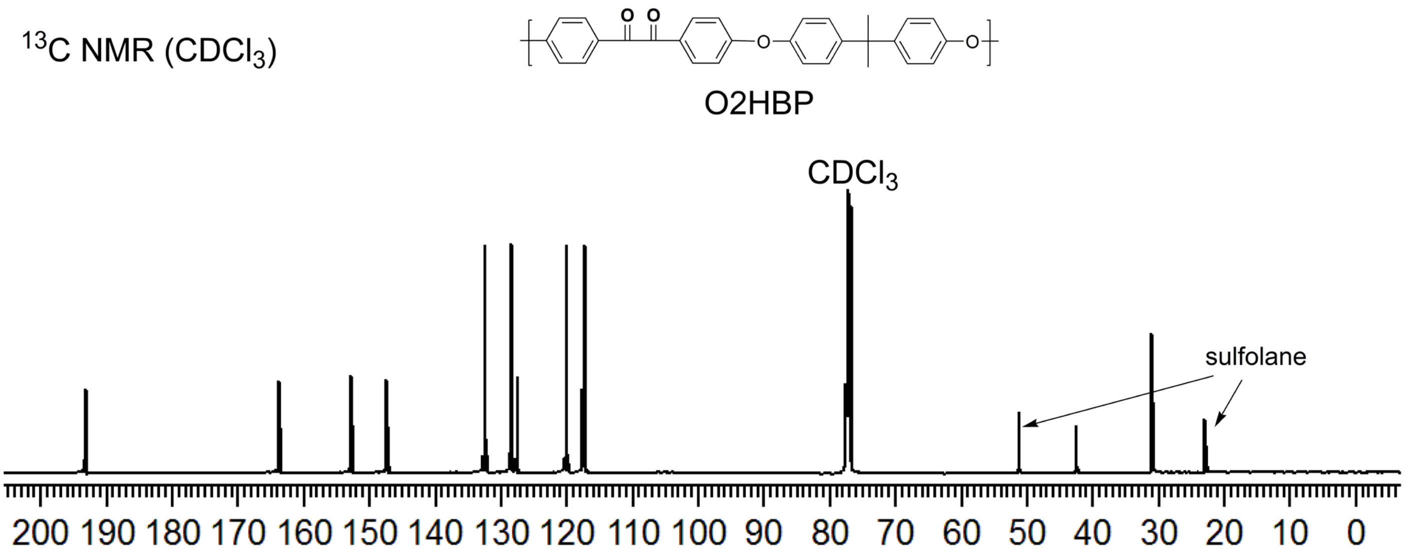

Figure S22. ^{13}C NMR spectrum of O2HBP.

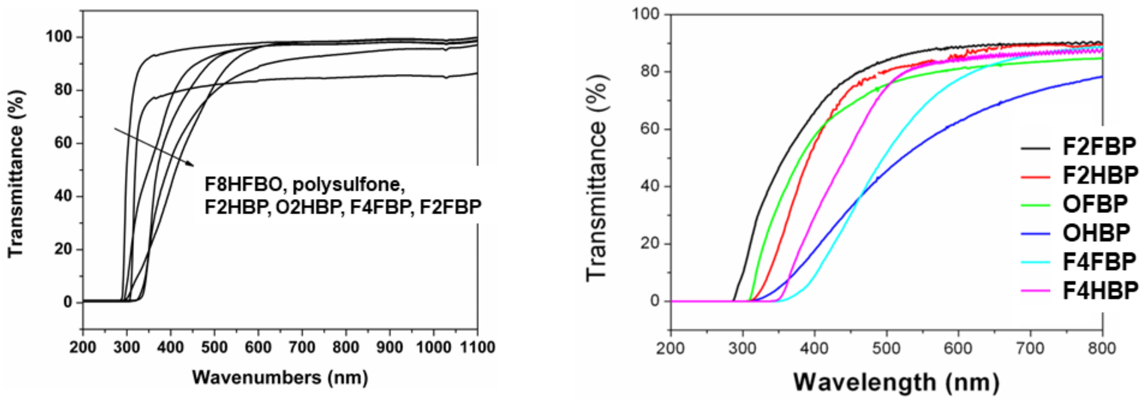

Figure S23. UV-visible spectrum of fluorinated polymers (F8HFBP was made as a comparison by following procedure of the following references: Goodwin, A. A.; Mercer, F. W., McKenzie, M. T. *Macromolecules* **1997**, *30*, 2767-2774; Song, Y.; Wang, J.; Li, G.; Sun, Q.; Jian, X.; Teng, J.; Zhang, H. *Polymer* **2008**, *49*, 4995-5001.).

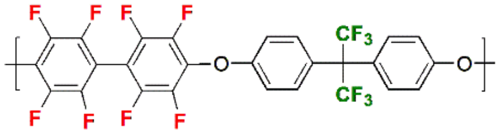

reference:
Macromolecules
1997, *30*, 2767

F8HFBP

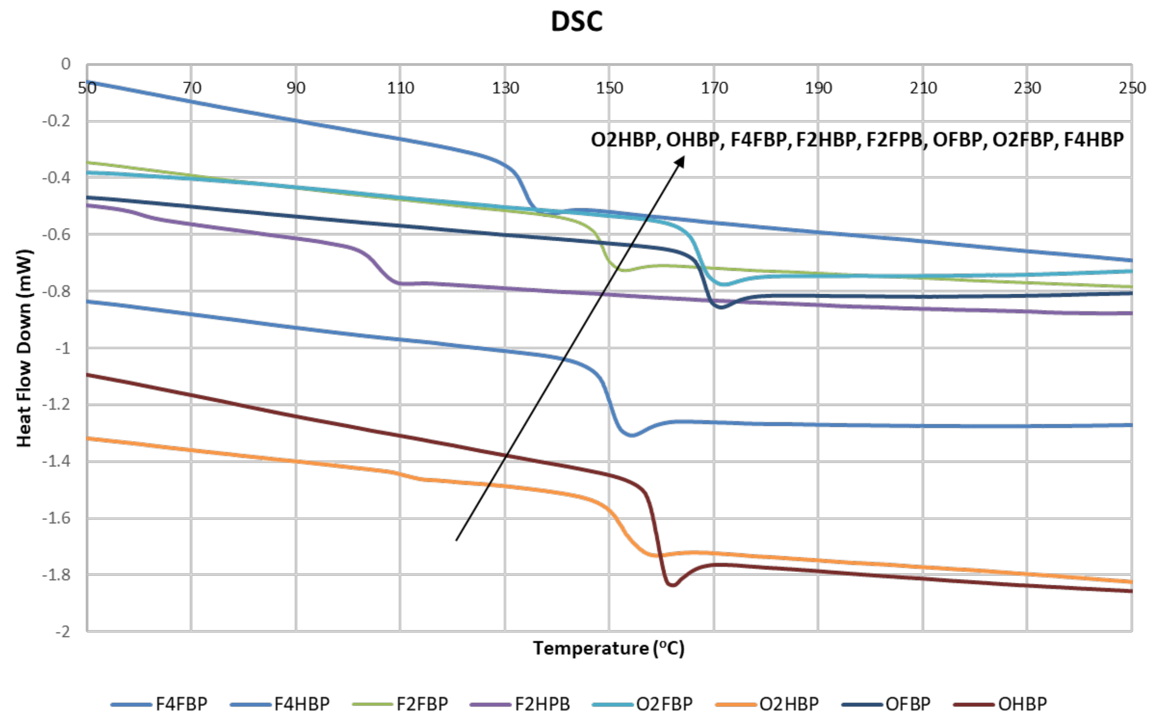

Figure S24. DSC curves of poly(aryl ether)s.

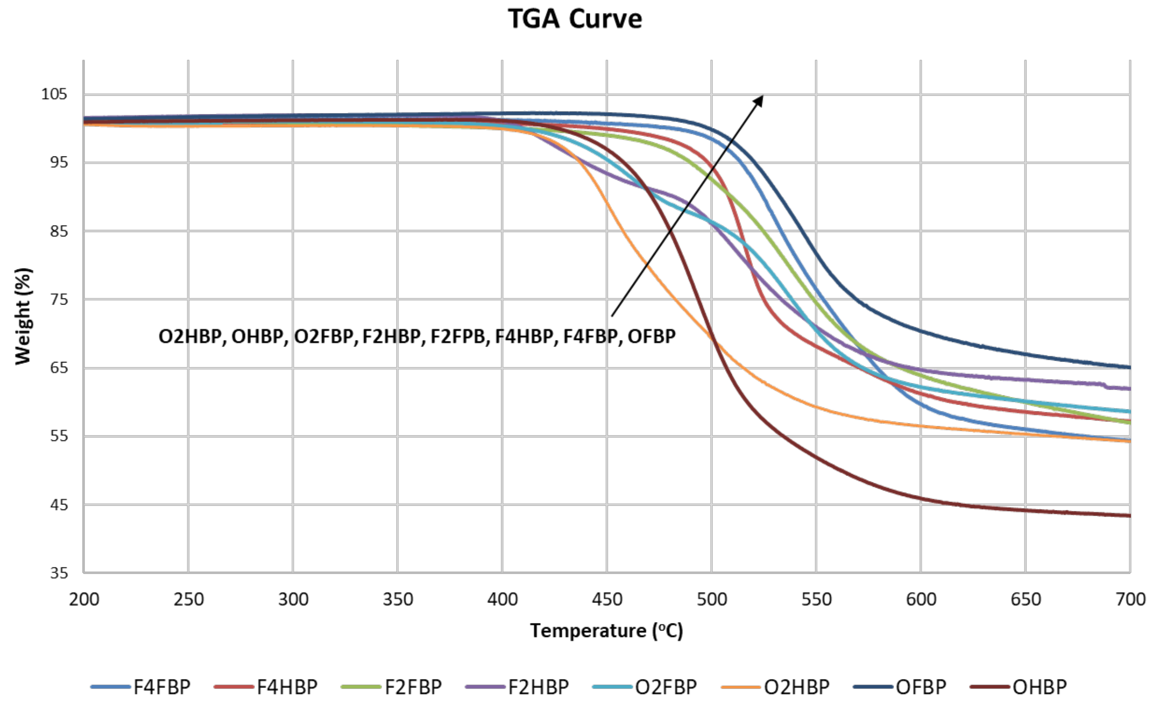

Figure S25. TGA curves of poly(aryl ether)s.
